# Nuclear translocation of PLSCR1 activates STAT1 signaling in basal-like breast cancer

**DOI:** 10.7150/thno.43150

**Published:** 2020-03-25

**Authors:** Panpan Huang, Ruocen Liao, Xingyu Chen, Xuebiao Wu, Xiaoli Li, Yifan Wang, Qianhua Cao, Chenfang Dong

**Affiliations:** 1 Department of Pathology and Pathophysiology, and Department of Surgical Oncology (breast center) of the Second Affiliated Hospital, Zhejiang University School of Medicine, Hangzhou 310058, China.; 2 Zhejiang Key Laboratory for Disease Proteomics, Zhejiang University School of Medicine, Hangzhou 310058, China.; 3 Department of Pathophysiology, Zunyi Medical University, Zunyi 563000, China; 4 Cancer Institute of Integrative Medicine, Zhejiang Academy of Traditional Chinese Medicine, Tongde Hospital of Zhejiang Province, Hangzhou, China.

**Keywords:** PLSCR1, basal-like breast cancer, STAT3, STAT1 signaling, cancer stem cells

## Abstract

**Rationale:** Basal-like breast cancer (BLBC) is associated with high grade, distant metastasis, and poor prognosis; however, the mechanism underlying aggressiveness of BLBC is still unclear. Emerging evidence has suggested that phospholipid scramblase 1 (PLSCR1) is involved in tumor progression. Here, we aimed to study the possible involvement and molecular mechanisms of PLSCR1 contributing to the aggressive behavior of BLBC.

**Methods:** The potential functions of PLSCR1 in breast cancer cells were assessed by Western blotting, colony formation, migration and invasion, Cell Counting Kit-8 assay, mammosphere formation and flow cytometry. The relationship between nuclear translocation of PLSCR1 and transactivation of STAT1 was examined by immunostaining, co-IP, ChIP, and quantitative reverse transcription PCR. The effect of PLSCR1 expression on BLBC cells was determined by *in vitro* and *in vivo* tumorigenesis and a lung metastasis mouse model.

**Results:** Compared to other subtypes, PLSCR1 was considerably increased in BLBC. Phosphorylation of PLSCR1 at Tyr 69/74 contributed to the nuclear translocation of this protein. PLSCR1 was enriched in the promoter region of STAT1 and enhanced STAT3 binding to the STAT1 promoter, resulting in transactivation of STAT1; STAT1 then enhanced cancer stem cell (CSC)-like properties that promoted BLBC progression. The knockdown of PLSCR1 led to significant inhibitory effects on proliferation, migration, invasion, tumor growth and lung metastasis of BLBC cells. Clinically, high PLSCR1 expression was strongly correlated with large tumor size, high grade, metastasis, chemotherapy resistance, and poor survival, indicating poor prognosis in breast cancer patients.

**Conclusions:** Our data show that overexpression and nuclear translocation of PLSCR1 provide tumorigenic and metastatic advantages by activating STAT1 signaling in BLBC. This study not only reveals a critical mechanism of how PLSCR1 contributes to BLBC progression, but also suggests potential prognostic indicators and therapeutic targets for this challenging disease.

## Introduction

Phospholipid scramblase 1 (PLSCR1) is a member of the family of membrane proteins that mediate the transbilayer movement of phospholipids (“scrambling”) in a Ca^2+^ dependent manner 1-3. However, the function of PLSCR1 as a scramblase has been challenged. For example, PLSCR1 overexpression did not increase the externalization of phospholipids in several cell lines, and PLSCR1-knockout mice had no alteration of PLSCR1 scrambling 4, 5. These reports suggest that besides phospholipid scrambling, PLSCR1 might have other roles in the cells.

A cysteine-rich palmitoylation motif [^184^CCCPCC^189^] of PLSCR1, a thioester linkage to the sulfhydryl groups of cysteines, controls the transportation of PLSCR1 to the cell membrane or nucleus 6. Nuclear PLSCR1 is observed when palmitoylation of the protein is prevented 7. It has been proposed that PLSCR1 interacts with multiple proteins such as ECM1 and EGFR that are involved in intracellular signaling pathways 7. Apart from binding to cellular proteins, PLSCR1 also has potential nuclear functions. A nonconventional nuclear localization signal (NLS) [^257^GKISKHWTGI^266^] present in PLSCR1 targets the protein to the nucleus by an energy-dependent pathway 7. Following the entry into the nucleus, PLSCR1 binds to the promoter region of the inositol 1,4,5-triphosphate receptor type 1 (IP3R1) gene, activating its transcription 8. However, it remains to be determined whether nuclear PLSCR1 also binds to other genes to influence their expressions.

Signal transducer and activator of transcription 1 (STAT1) is a transcription factor that is associated with the interferon pathway. In breast cancer, it is considered as a tumor suppressor as STAT1-deficient mice develop estrogen receptor (ER)-positive mammary carcinomas 9. However, several studies imply that STAT1 is a tumor promotor in various cancers 10, 11. It has been reported that STAT1 promotes breast cancer progression by increasing cancer stemness 11 and contributes to radioresistance in breast cancer-initiating cells 12.

Despite extensive studies, little is known about the functions and underlying mechanisms of PLSCR1 in tumor progression. In this study, we show that PLSCR1 expression is significantly upregulated in basal-like breast cancer (BLBC), a subtype that is associated with large tumor size, high grade, metastasis, early recurrence, and poor survival 13-15. We provide evidence that PLSCR1 enhances stem cell-like properties through activating STAT1 signaling in BLBC. Furthermore, our study elucidates a critical mechanism of how PLSCR1 is transported to the nucleus and contributes to tumor progression in BLBC.

## Material and Methods

### Plasmids, shRNA, and antibodies

PLSCR1 shRNA and STAT3 shRNA were purchased from Sigma-Aldrich (St Louis, MO). Human PLSCR1, STAT3, and EGFR were amplified from the MDA-MB231 cDNA and subcloned into pLenti6.3⁄V5, pLVX, and pCMV, respectively.

Recombinant human EGF protein, human interleukin-6 (hIL-6), and antibodies against PLSCR1 and ALDH1 were purchased from Abcam (Carlsbad, CA). Antibodies against STAT1, STAT3, Phospho- STAT1 (Tyr701) and Phospho-STAT3 (Tyr705) were acquired from Cell Signaling Technology (Danvers, MA). Antibodies against phospho-Tyr were obtained from Santa Cruz (Dallas, TX). 6x-His Tag monoclonal antibody and Alexa Fluor 555-conjugated goat anti-rabbit IgG were purchased from Thermo Fisher Scientific. Antibodies for Flag, Myc, β-Tubulin, LaminB and Anti-FLAG Magnetic Beads were obtained from Sigma-Aldrich (St. Louis, MO).

### Cell culture

All cells we used in this study were obtained from the American Type Culture Collection (Manassas, VA), where the cell lines were authenticated by STR profiling before distribution. The cells were cultured and stored according to the supplier's instructions. After resuscitation, cells were grown in the medium with 10% fetal bovine serum (FBS), never passaged longer than 6 months and tested routinely by Hoechst DNA staining to ensure no mycoplasma contamination. MDA-MB231, SUM159, and MCF7 cells were grown in Dulbecco's modified Eagle's Medium (DMEM)/F12 supplemented with 10% FBS. HCC1937 and T47D cells were grown in RPMI1640 plus 10% FBS. MDA-MB468 cells were cultured in Leibovitz's L-15 medium supplemented with 10% FBS. For establishing stable transfectants with PLSCR1 expression or knockdown of PLSCR1 expression, luminal cells and BLBC cells were transfected with pLenti6.3⁄V5-PLSCR1 and PLSCR1 shRNA, respectively; stable clones were selected with blasticidin (2 µg/ml) and puromycin (300 ng/ml) for 4 weeks, respectively.

### Immunostaining

Experiments were performed as described previously 16. Cells grown on chamber slides were fixed for 15 min with 4% paraformaldehyde, permeabilized for 10 min in phosphate-buffered saline (PBS) containing 0.2% TritonX-100, blocked for 1 h with 1% BSA and 0.5% goat serum in PBS, and then incubated with primary antibodies at 4°C overnight. After rinsing with PBS, the cells were incubated with secondary antibodies for 1 h at room temperature and the nuclei were stained with DAPI (Sigma) for 5 min. Alexa Fluor 555-conjugated goat anti-rabbit antibodies (Molecular Probes) were used as secondary antibodies. Following three washes with HBSS, fluorescence was examined by an Olympus Confocal Laser Scanning Microscope (OLYMPUS IX83-FV3000-OSR).

### Quantitative Real-Time PCR

Total RNA was extracted from cells by RNeasy Mini kit (Qiagen) according to the manufacturer's instructions. Reverse transcription was performed with the QuantiTect Reverse Transcription Kit (Qiagen). Specific quantitative real-time PCR was performed using SYBR Green Power Master Mix according to the manufacturer's protocol (Applied Biosystems). Gene expression level was normalized to actin level in respective samples as an internal control, and the results were representative of at least three independent experiments.

### Chromatin Immunoprecipitation (ChIP)

The primers used for ChIP assays were: 5'-CACGGAGGTCAGTTGCTAAA-3′ (forward) and 5'-AGAAGGACGTGCTGTGTTTG-3′ (reverse) for the STAT1 promoter; 5'-ACTCAGTCTGGGTGGAAGGTATC-3′ (forward) and 5'-AGATAGGGAGGAATGATAGAGGC-3′ (reverse) for the c-Myc promoter. The cells were prepared to perform ChIP assay with the ChIP Kit (Cell Signaling Technology) according to the manufacturer's instructions as described previously 17, 18.

### Immunoblotting of tumor samples

The breast tumor samples were collected from patients with informed consent. The experiments were performed according to the guidelines approved by the Institutional Review Board at Zhejiang University (Hangzhou, China). The samples were homogenized in 1 ml of homogenizing buffer. The extracted proteins were boiled and analyzed by SDS-PAGE, and then transferred onto PVDF membranes (Thermo Fisher Scientific). Immunoreactive bands were examined by chemiluminescence.

### Flow cytometry

Cells were washed and suspended in 1 mL PBS, and then 1×10^6^ cells were incubated with monoclonal antibodies CD44-APC and CD24-PE (eBiosciences) at 4℃ for 30min in the dark according to the manufacturer's instructions. Following two washes with PBS, the cells were analyzed by ACEA NovoCyteTM.

### Colony formation assay

Colony formation assay was performed using double-layer soft agar in 24-well plates with a bottom layer of 0.7% agar and a top layer of 0.35% agar. Cells were seeded in 24-well plates and cultured at 37°C for 15-20 days, and the colonies were counted as described previously 19.

### Migration, invasion, and mammosphere assays

Migration and invasion assays were carried out as described previously 20. All experiments were repeated at least twice in triplicate. Statistical analysis was done using the Student's t-test. Mammosphere assays were performed according to the protocol described previously 21 by planting single-cell suspension into ultralow-attachment 6-well plates (Corning Life Sciences) in the mammosphere culturing condition and counted after 10 to 15 days.

### Tumorigenesis assay and lung metastasis model

Animal experiments were performed according to the approved procedures by the Institutional Animal Care and Use Committee at Zhejiang University. To test the effect of PLSCR1 on *in vivo* tumorigenesis, female SCID mice (5-7 wks old) were injected with 1×10^6^ exogenous PLSCR1 knockdown cells in the left flank and vector control cells in the right flank. Tumor formation and growth were monitored every 2 days for 30 days, and tumor size and weight were determined. To evaluate the effect of PLSCR1 on tumor lung metastasis, SCID mice were injected via tail vein with MDA-MB231 cells (1x10^6^ cells/mouse) with stable empty vector or knockdown of PLSCR1 expression (6 mice/group). After 4 weeks, lung metastasis was examined by an IVIS-100 imagining system (Xenogen). Lung metastatic nodules were analyzed in paraffin-embedded sections stained with hematoxylin and eosin. Data analyses were performed using the Student's t-test; a p-value <0.05 was considered significant.

### Statistical analysis

Results were expressed as mean ± SD or SEM as indicated. Comparisons were made by one-way ANOVA or the two-tailed Student's t-test. Correlations between STAT1 and PLSCR1 were determined by Pearson's correlation and Spearman's rank correlation test. Survival curves were analyzed using the Kaplan-Meier method, and differences were compared by the log-rank test. In all statistical tests, p < 0.05 was considered statistically significant.

## Results

### PLSCR1 is overexpressed in BLBC subtype

**Figure 1:**
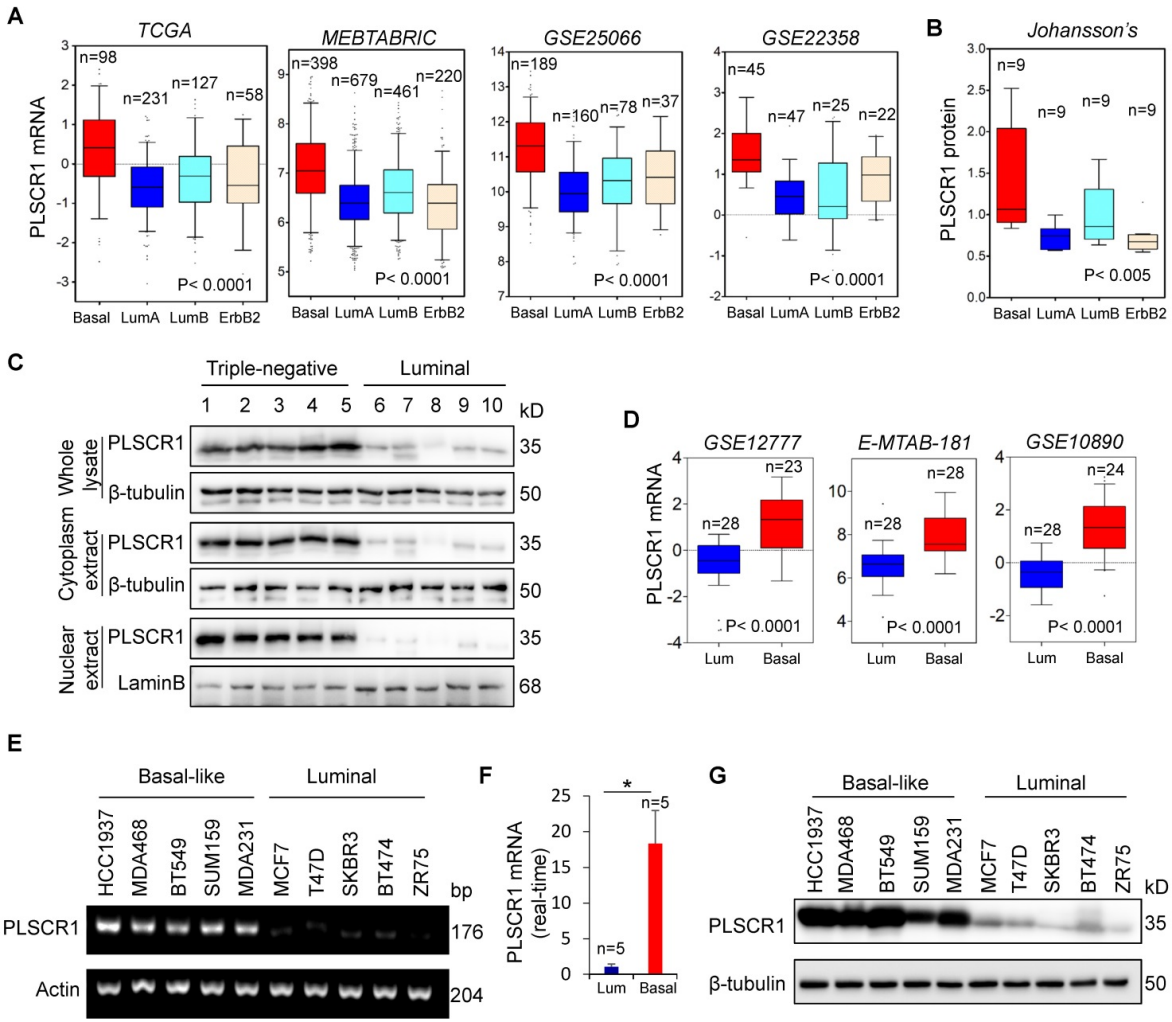
** Elevated PLSCR1 expression tightly correlates with BLBC. (A)** Box-plots indicate PLSCR1 mRNA expression in breast cancer from four datasets (TCGA, MEBTABRIC, GSE25066, and GSE22358). **(B)** Box-plots indicate PLSCR1 protein expression in breast cancer from the Johansson's dataset. **(C)** Expression of PLSCR1 was analyzed by Western blotting in five luminal and five triple-negative breast cancer samples. **(D)** Box-plots indicate PLSCR1 mRNA expression in luminal and BLBC cell lines from three datasets (GSE12777, E-MTAB-181 and GSE10890). **(E-F)** Expression of PLSCR1 mRNA was examined by either semi-quantitative RT-PCR (E) or quantitative real-time PCR (F) in breast cancer cell lines. *p< 0.05 by Student's t-test. **(G)** Expression of PLSCR1 in cells from (E) was analyzed by Western blotting.

We recently reported that several enzymes such as aldo-keto reductase 1 member B1 (AKR1B1), UDP- galactose ceramide galactosyltransferase (UGT8), and 4-aminobutyrate aminotransferase (ABAT) were closely related to BLBC aggressiveness 16, 20, 21. To further investigate other enzymes involved in BLBC, we analyzed multiple gene expression datasets (TCGA, MEBTABRIC, GSE25066, GSE22358, NKI295, and GSE7390) that contain over 4000 breast cancer patients 22-25. Besides some previously identified genes, such as fructose-1, 6-biphosphatase (FBP1) and AKR1B1 26, PLSCR1 mRNA expression that associates with both lipid trafficking and cell signaling was dramatically elevated in BLBC (**Figure 1A** and **Figure S1A**).

We analyzed a proteogenomic dataset containing 36 breast tumor samples 27, and found PLSCR1 protein expression to be significantly higher in BLBC than in other subtypes (**Figure 1B**). To confirm this observation, we analyzed PLSCR1 levels in both the nucleus and cytoplasm of breast tumor tissues. Consistently, PLSCR1 protein level was much higher in triple-negative breast cancer (TNBC), which mostly overlaps with BLBC, than in luminal subtype of breast cancers (**Figure 1C**). To further verify the association of PLSCR1 with the basal subtype, we also examined PLSCR1 expression in five gene expression datasets (GSE12777, E-MTAB-181, GSE10890 and GSE16732) that contain 51, 56, 52 and 41 breast cancer cell lines, respectively 28-31. Strikingly, PLSCR1 expression was upregulated in BLBC cell lines (**Figure 1D** and **Figure S1B**). We then confirmed this observation by either semi-quantitative RT-PCR or qRT-PCR in five luminal and five basal subtype cell lines and found that PLSCR1 mRNA expression was apparently higher in BLBC cells than in luminal cells (**Figure 1E-F**). We further examined PLSCR1 protein expression and detected an elevated level in BLBC cell lines (**Figure 1G**). These findings support that PLSCR1 overexpression positively correlates with the BLBC subtype.

### PLSCR1 expression enhances breast cancer cell proliferation, migration, and invasion

**Figure 2:**
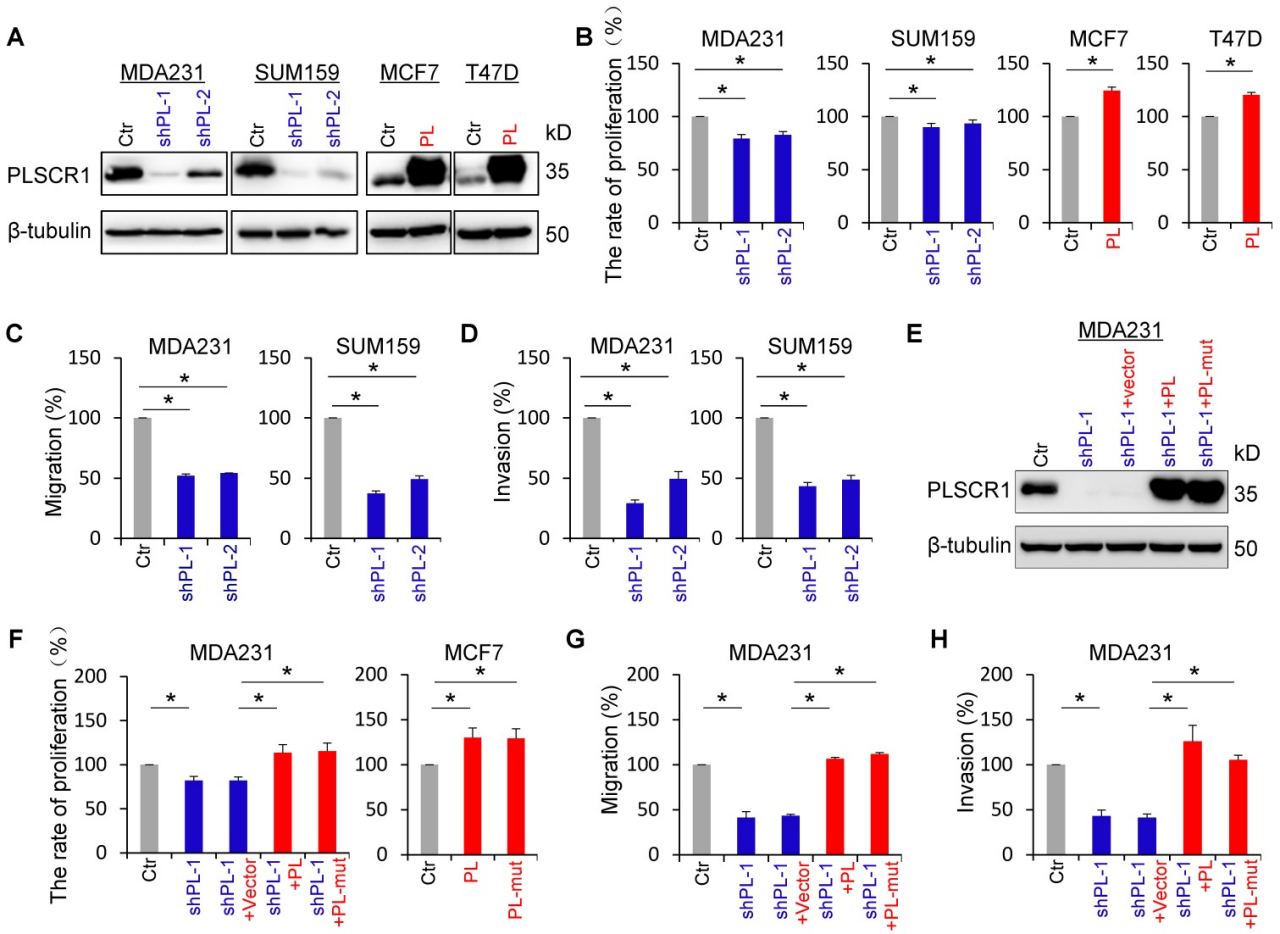
** PLSCR1 expression promotes breast cancer cell proliferation, migration, and invasion. (A)** Expression of PLSCR1 was examined by Western blotting in MDA-MB231 and SUM159 cells with stable empty vector or knockdown of PLSCR1 expression as well as MCF7 and T47D cells with stable empty vector or PLSCR1 expression. **(B)** Growth of MDA-MB231 and SUM159 cells with stable empty vector or knockdown of PLSCR1 expression (left panel) as well as MCF7 and T47D cells with stable empty vector or PLSCR1 expression (right panel) was analyzed by CCK-8 assay for 120 hours. Data are presented as a percentage over control cells (mean ± SD in three separate experiments). *p< 0.05 by Student's t-test. **(C-D)** Migration (C) and invasiveness (D) of MDA-MB231 and SUM159 cells with stable empty vector or knockdown of PLSCR1 expression were analyzed. The percentage of migratory and invasive cells are presented in the bar graph (mean ± SD in three separate experiments). *p< 0.01 by Student's t-test. **(E)** Expression of PLSCR1 was examined by Western blotting in MDA-MB231 cells with stable empty vector or knockdown of PLSCR1 expression as well as shPLSCR1-expressing MDA-MB231 cells with stable empty vector, PLSCR1, or PLSCR1-mut (no enzymatic activity) expression. **(F)** Cell growth of MDA-MB231 cells with stable empty vector or knockdown of PLSCR1 expression as well as shPLSCR1-expressing MDA-MB231 cells with stable empty vector, PLSCR1, or PLSCR1-mut expression (left panel), and MCF7 cells with stable empty vector, PLSCR1, or PLSCR1-mut expression (right panel) was analyzed by CCK-8 assay for a period of 120 hours. Data are presented as a percentage over control cells (mean ± SD in three separate experiments). *p< 0.05 by Student's t-test. **(G-H)** Migration (G) and invasiveness (H) of MDA-MB231 cells with stable empty vector or knockdown of PLSCR1 expression as well as shPLSCR1-expressing MDA-MB231 cells with stable empty vector, PLSCR1, or PLSCR1-mut expression were analyzed. The percentage of migratory and invasive cells is presented in the bar graph (mean ± SD in three separate experiments). *p< 0.01 by Student's t-test.

To explore the molecular function and mechanism of PLSCR1, we established stable transfectants with empty vector or knockdown of PLSCR1 expression in MDA-MB231 and SUM159 cells, and with empty vector or PLSCR1 expression in MCF7 and T47D cells (**Figure 2A**). We first analyzed the effect of PLSCR1 expression on breast cancer cell proliferation, and found that knockdown of PLSCR1 expression caused a slight but significant decrease in MDA-MB231 and SUM159 cell proliferation. On the contrary, exogenous PLSCR1 expression led to a significant increase in the proliferation of MCF7 and T47D cells (**Figure 2B**). Analysis of the effect of PLSCR1 expression on breast cancer cell migration and invasion showed that knockdown of PLSCR1 expression markedly repressed the migration and invasion of MDA-MB231 and SUM159 cells (**Figure 2C-D**). It has been reported that aspartate to alanine mutations in the segment ^273^DADNFGIQFPLD^284^ result in loss of calcium binding and scramblase activity 32. We, therefore, generated the PLSCR1- mut expression plasmid, expressed PLSCR1 and PLSCR1-mut that loses enzymatic activity in MCF7, T47D, and shPLSCR1-expressing MDA-MB231 and SUM159 cells, and examined the effect of PLSCR1 expression and enzymatic activity on breast cancer cell proliferation, migration and invasion. Significantly, both PLSCR1 and PLSCR1-mut expression caused a similar level of increase in the proliferation of MCF7 and T47D cells (**Figure 2F** and **Figure S2B**). Additionally, either PLSCR1 or PLSCR1-mut expression restored the decreased proliferation, migration and invasion of MDA-MB231 and SUM159 cells with stable knockdown of PLSCR1 expression (**Figure 2E-H** and **Figure S2A-C**). These data indicate that the expression of PLSCR1, not its enzymatic activity, is responsible for controlling proliferation, migration, and invasion of breast cancer cells.

### Phosphorylation of PLSCR1 contributes to its nuclear translocation

**Figure 3:**
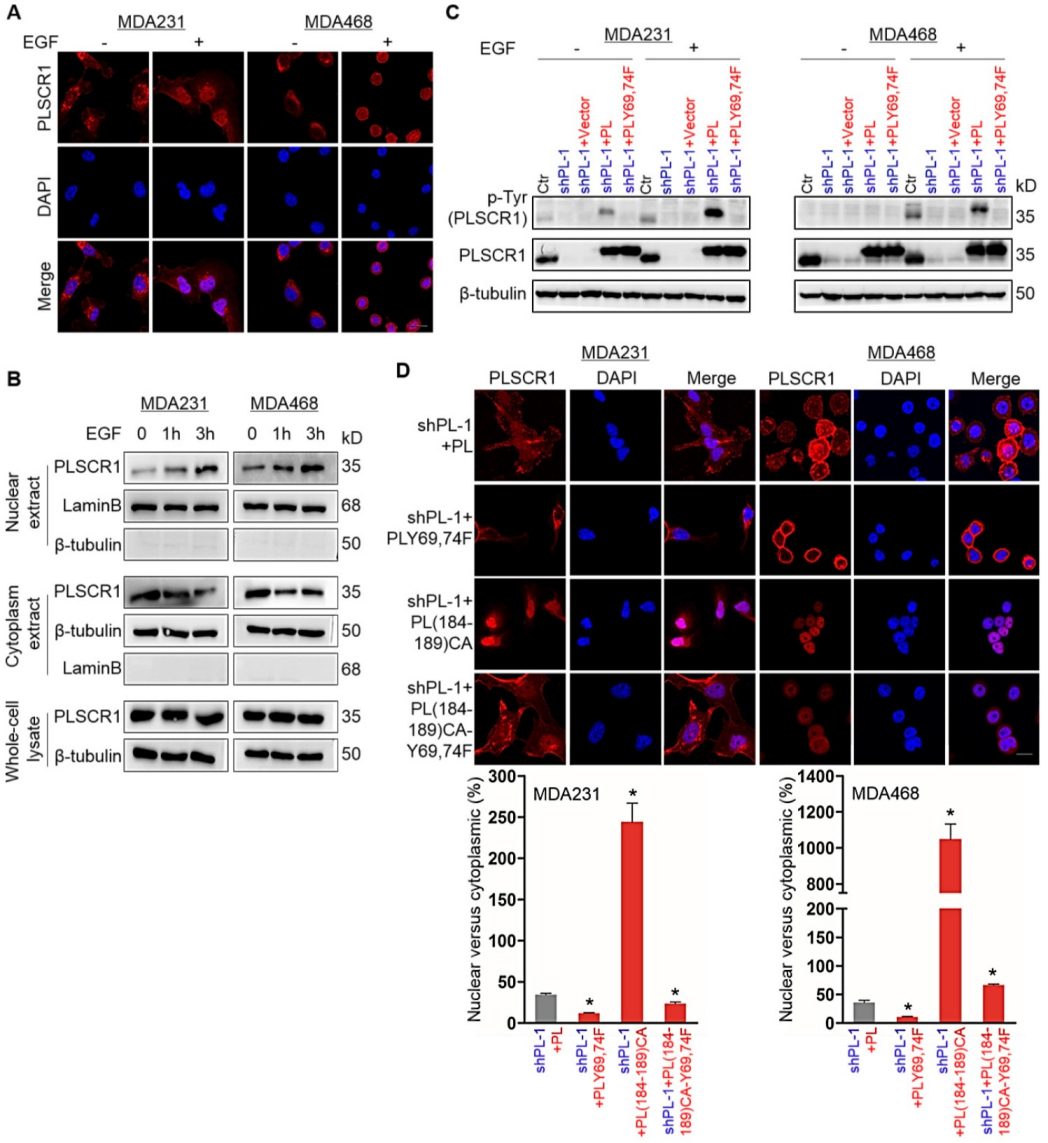
** Phosphorylation of PLSCR1 contributes to the nuclear translocation of the protein. (A)** Expression of PLSCR1 was measured by immunofluorescent staining in MDA-MB231 and MDA-MB468 cells treated with or without EGF (100 ng/ml). Nuclei were visualized with DAPI (blue). Scale bar = 20 μm (right). **(B)** Expression of PLSCR1 was examined by Western blotting in MDA-MB231 and MDA-MB468 cells treated with or without EGF (100 ng/ml) for a period of 0, 1, or 3 hours. **(C)** Expression of PLSCR1 was examined by Western blotting in MDA-MB231 and MDA-MB468 cells with stable empty vector or knockdown of PLSCR1 expression as well as shPLSCR1-expressing MDA-MB231 and MDA-MB468 cells with stable empty vector, PLSCR1, or PLSCR1-Y69, 74F expression following treatment with or without EGF (100 ng/ml). **(D)** Expression and localization of PLSCR1 were measured by immunofluorescent staining in shPLSCR1-expressing MDA-MB231 and MDA-MB468 cells with stable PLSCR1, PLSCR1-Y69, 74F, PLSCR1 (184-189) CA, or PLSCR1 (184-189) CA-Y69, 74F expression following treatment with EGF (100 ng/ml) (top panel). Nuclei were visualized with DAPI (blue). Scale bar = 20 μm (right). The nuclear-cytoplasmic staining percentage is shown in the bottom panel (mean ± SD in three separate experiments). *p< 0.01 by Student's t-test.

PLSCR1 is localized to the cell membrane, cytoplasm and nucleus; however under normal growth conditions only a small percentage of PLSCR1 is detected in cell nuclei 3. PLSCR1 has also been reported to interact with EGFR in EGF-stimulated epidermoid carcinoma cells 7. To investigate the effect of EGFR signaling on PLSCR1 distribution, we examined the subcellular localization of PLSCR1 following EGF stimulation in MDA-MB231, MDA- MB468, and HCC1937 cells. Immunostaining-confocal analysis showed that EGF treatment dramatically enhanced nuclear translocation of PLSCR1 in three cell lines (**Figure 3A** and **Figure S3A**). To further analyze the nuclear translocation of PLSCR1, we examined cytosolic and nuclear fractions by Western blotting. Consistently, EGF treatment led to a marked increase of endogenous PLSCR1 in a time-dependent manner in the nuclear extracts of these cell lines (**Figure 3B**; and **Figure S3B**). These data indicate that EGF-mediated signaling is required for nuclear translocation of PLSCR1.

It has been shown that EGF signaling mediates phosphorylation on tyrosines 69 and 74 (Tyr^69^ and Tyr^74^) of PLSCR1 33. We speculated that EGF- mediated phosphorylation of PLSCR1 Tyr69 and Tyr74 might be involved in nuclear translocation PLSCR1. To test this notion, we examined PLSCR1 phosphorylation after EGF stimulation by Western blotting. We expressed PLSCR1 and PLSCR1-Y69, 74F mutation (PLSCR1-Y69, 74F) in shPLSCR1-expressing MDA-MB231 and MDA-MB468 cells (**Figure 3C**). Following EGF stimulation, exogenetic PLSCR1, but not PLSCR1-Y69, 74F expression remarkably restored the decreased PLSCR1 phosphorylation in these cells with stable knockdown of PLSCR1 expression (**Figure 3C**). This finding was further validated by immunostaining-confocal analysis (**Figure 3D** and **Figure S3C**). The cysteine rich palmitoylation motif [^184^CCCPCC^189^] of PLSCR1 is a checkpoint that determines the location of this protein between the cell membrane and nucleus, and non-palmitoylated PLSCR1 is released into the cytosol and subsequently transported into the nucleus 6. We thus expressed mutant ^184^AAAPAA^189^ PLSCR1 [PLSCR1 (184-189) CA] that cannot be palmitoylated and PLSCR1 (184-189) CA with Y69, 74F [PLSCR1 (184-189) CA-Y69, 74F] in shPLSCR1-expressing MDA-MB231, MDA-MB468, and HCC1937 cells (**Figure 3D** and **Figure S3C**). After EGF treatment, mutant^ 184^AAAPAA^189^ contributed to nuclear translocation of PLSCR1 compared with wild-type ^184^CCCPCC^189^, whereas mutant Y69, 74F efficiently blocked mutant ^184^AAAPAA^189^ -induced nuclear translocation of PLSCR1 (**Figure 3D** and **Figure S3C**). These findings suggest that phosphorylation of PLSCR1 Tyr69 and Tyr74 is required for EGF-induced PLSCR1 nuclear translocation.

### PLSCR1 positively correlates with STAT1

**Figure 4:**
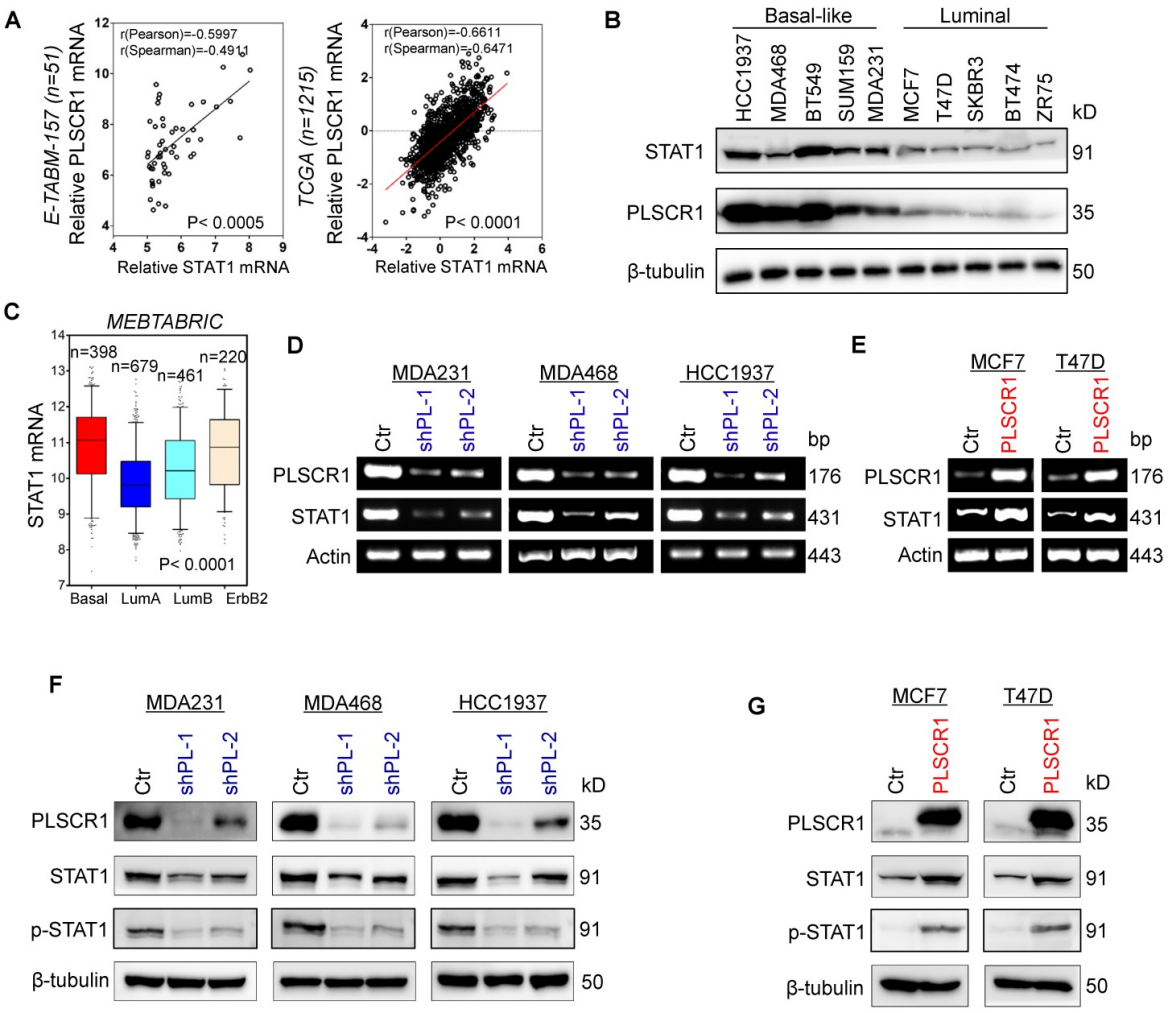
** PLSCR1 positively correlates with STAT1. (A)** Analysis of E-TABM-157 and TCGA datasets for the expression of PLSCR1 and STAT1. The relative level of PLSCR1 is plotted against that of STAT1. **(B)** Expression of PLSCR1 and STAT1 was examined by Western blotting in breast cancer cell lines. **(C)** Box-plots indicate STAT1 mRNA expression in breast cancer from the MEBTABRIC dataset. **(D-E)** Expression of PLSCR1 and STAT1 mRNA was examined by semi-quantitative RT-PCR in MDA-MB231, MDA-MB468, and HCC1937 cells with stable empty vector or knockdown of PLSCR1 expression (D) as well as MCF7 and T47D cells with stable empty vector or PLSCR1 expression (E). **(F-G)** Expression of PLSCR1, STAT1, and p-STAT1 was examined by Western blotting in MDA-MB231, MDA-MB468, and HCC1937 cells with stable empty vector or knockdown of PLSCR1 expression (F) as well as MCF7 and T47D cells with stable empty vector or PLSCR1 expression (G).

To explore potential molecular mechanisms of PLSCR1 in breast cancer, we investigated the correlation of PLSCR1 with other proteins. Co-expression analysis of PLSCR1 with other genes in a gene expression dataset (E-TABM-157) that contains 51 breast cancer cell lines showed that PLSCR1 expression positively correlated with STAT1 expression (**Figure 4A**). A similar result was observed in analyzing another gene expression dataset (TCGA) that has 1215 breast cancer patients (**Figure 4A**). We further examined the expression of PLSCR1 and STAT1 in five luminal and five BLBC cell lines and found their expression to be elevated in BLBC and decreased in luminal cell lines (**Figure 4B**), supporting the correlation between PLSCR1 and STAT1. Analysis of STAT1 expression in different subtypes of breast cancer showed that, similar to PLSCR1, STAT1 was significantly upregulated in BLBC in the MEBTABRIC dataset (**Figure 4C** and **Figure 4A**). We then explored the causal relationship between PLSCR1 and STAT1. Significantly, knockdown of PLSCR1 expression downregulated, whereas endogenous PLSCR1 expression upregulated, STAT1 expression and phosphorylated STAT1 levels (**Figure 4D-G**). These data indicate a critical role of PLSCR1 in regulating STAT1 expression.

### PLSCR1 interacts with STAT3

**Figure 5:**
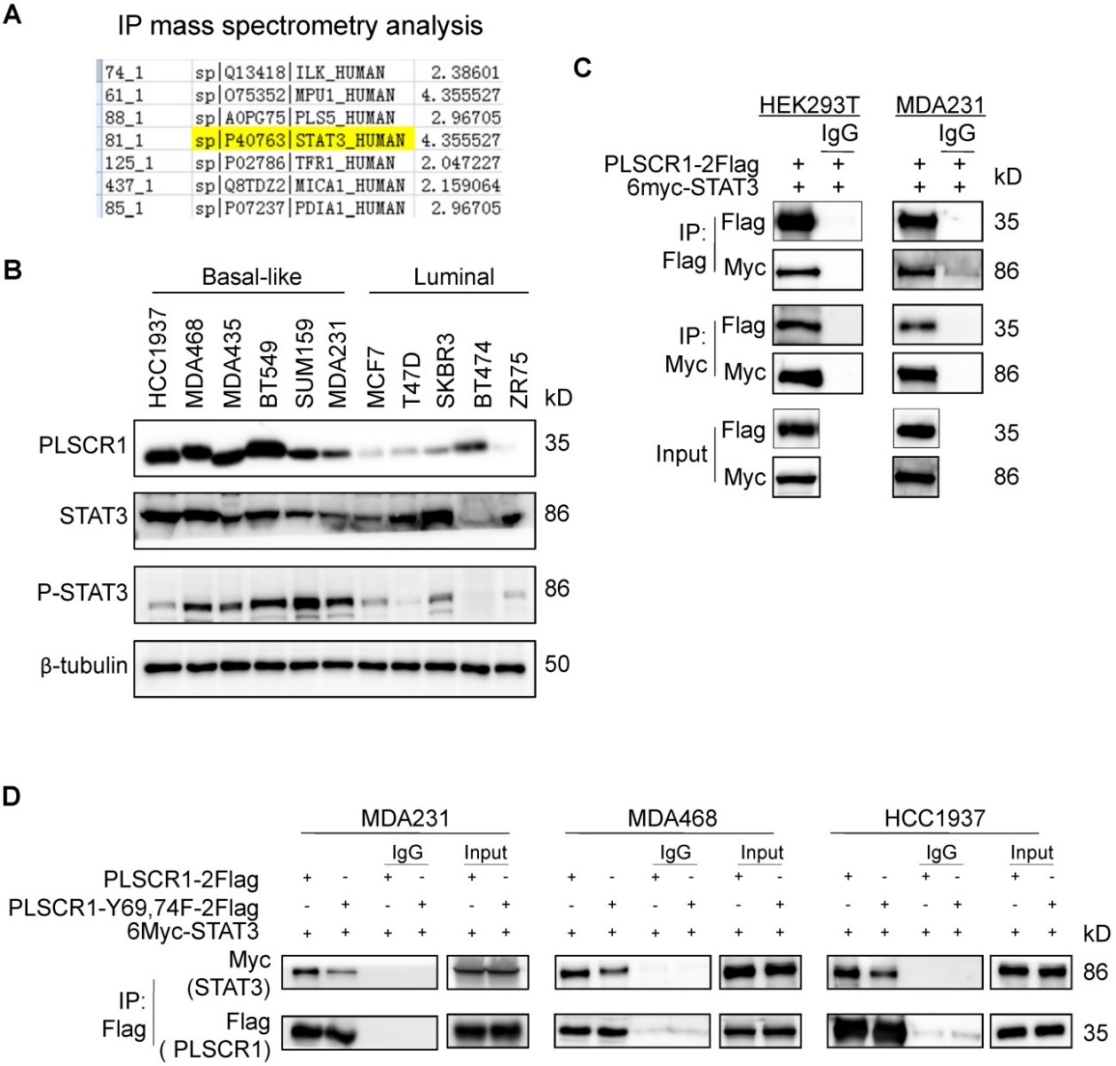
** PLSCR1 interacts with STAT3. (A)** MDA-MB231 cells with stable shPLSCR1-expression and PLSCR1-2Flag expression were established, and the PLSCR1 complex was isolated by Flag affinity columns. The bound proteins, such as STAT3, were identified by mass spectrometry. **(B)** Expression of PLSCR1, STAT3, and p-STAT3 was examined by Western blotting in breast cancer cell lines. **(C)** PLSCR1-2Flag and 6myc-STAT3 were co-expressed in HEK293T and MDA-MB231 cells. Following immunoprecipitation, the bound STAT3 and PLSCR1 were examined by Western blotting. **(D)** 6myc-STAT3 and PLSCR1-2Flag or PLSCR1-Y69, 74-F-2Flag were co-expressed in MDA-MB231, MDA-MB468, and HCC1937 cells. Following immunoprecipitation, the bound STAT3, PLSCR1, and PLSCR1-Y69, 74F were examined by Western blotting.

Given the link between PLSCR1 and STAT1, we next determined the mechanism of STAT1 upregulation by PLSCR1 in BLBC. To identify the potential proteins that interact with PLSCR1, we created a stable shPLSCR1-expressing MDA-MB231 cell line expressing Flag-tagged PLSCR1 (PLSCR1-2Flag). Following enrichment of the cell extracts, we carried out protein purification with Flag affinity columns, and then the bound proteins were subjected to mass spectrometry analysis. Two known proteins, EGFR and FYN that interact with PLSCR1, were found in the complexes and thus validated the specificity of this system (**Figure S4A**). Interestingly, STAT3 was also identified as a protein associated with PLSCR1 (**Figure 5A**). We then analyzed the expressions of PLSCR1, STAT3, and phosphorylated STAT3 (p-STAT3) in breast cancer cell lines and observed that PLSCR1 expression positively correlated with p-STAT3 expression (**Figure 5B**).

To validate the physical interaction of PLSCR1 with STAT3, we co-expressed PLSCR1-2Flag and Myc-tagged STAT3 (6myc-STAT3) in HEK293T and MDA-MB231 cells and then performed a co-immunoprecipitation experiment. Following immunoprecipitation of PLSCR1, we detected the associated STAT3, and vice versa (**Figure 5C**), validating their interaction. Similarly, we also identified the interaction between PLSCR1 and EGFR (**Figure S4B**). Next, we investigated whether phosphorylated sites of PLSCR1 were associated with the binding of PLSCR1 to STAT3 by co-expressing 6myc-STAT3 and PLSCR1-2Flag or Flag-tagged PLSCR1-Y69, 74F in MDA-MB231, MDA-MB468, and HCC1937 cells. As shown in Figure 5D, mutant Y69, 74F efficiently weakened the interaction between STAT3 and PLSCR1, indicating that phosphorylation of PLSCR1 Tyr69 and Tyr74 is involved in the binding of PLSCR to STAT3.

### Nuclear PLSCR1 regulates STAT3-mediated STAT1 expression and contributes to the maintenance of CSCs

**Figure 6:**
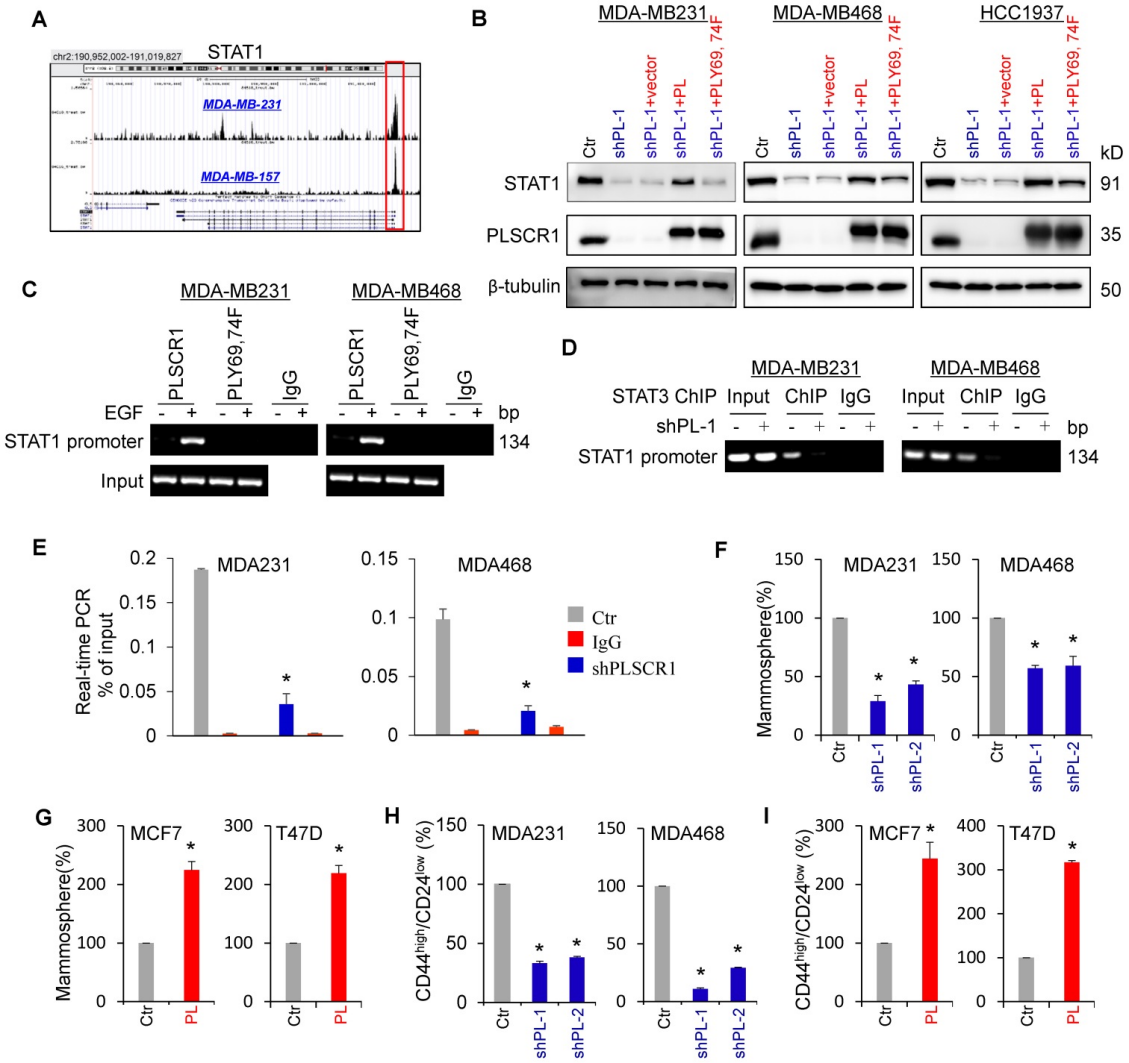
** Nuclear PLSCR1 regulates STAT3-mediated STAT1 expression and CSC properties. (A)** Read distribution and genomic localization around the promoter of the STAT1 gene identified as a direct target of STAT3. **(B)** Expression of PLSCR1 and STAT1 was examined by Western blotting in MDA-MB231, MDA-MB468, and HCC1937 cells with stable empty vector or knockdown of PLSCR1 expression as well as shPLSCR1-expressing cells with stable empty vector, PLSCR1, or PLSCR1-Y69, 74F expression. **(C)** Association of wild-type PLSCR1 or PLSCR1-Y69, 74F with the STAT1 promoter in MDA-MB231 and MDA-MB468 cells was analyzed by ChIP following treatment with or without EGF (100 ng/ml). **(D-E)** ChIP analysis for STAT3 binding to the STAT1 promoter in MDA-MB231 and MDA-MB468 cells with stable empty vector or knockdown of PLSCR1 expression following treatment with hIL-6 (100 ng/ml) by either semi-quantitative RT-PCR (D) or quantitative real-time PCR (E). Results from three independent experiments are presented (mean ± SD from three separate experiments). *p< 0.01 by Student's t-test. **(F-G)** Tumorsphere-formation of MDA-MB231 and MDA-MB468 cells with stable empty vector or knockdown of PLSCR1 expression (F) as well as of MCF7 and T47D cells with stable empty vector or PLSCR1 expression (G) was measured. Data are shown as a percentage of control cell lines (mean ± SD in three separate experiments). *p< 0.01 by Student's t-test. **(H-I)** Population of CSCs (CD44^high^/CD24^low^) were analyzed by flow cytometry in MDA-MB231 and MDA-MB468 cells with stable empty vector or knockdown of PLSCR1 expression (H) as well as in MCF7 and T47D cells with stable empty vector or PLSCR1 expression (I). Data are presented as a percentage of control cell lines as in (F). *p< 0.01 by Student's t-test.

STAT1 has been identified as a direct target gene of STAT3 34. Indeed, STAT3 was observed to be highly enriched in the promoter of STAT1 in MDA-MB231 and MDA-MB157 cells by previous STAT3-specific ChIP-seq analysis (**Figure 6A**) 35. Consistent with this observation, knockdown of STAT3 expression inhibited, whereas exogenous STAT3 expression upregulated STAT1 expression (**Figure S5A-B**). Because phosphorylation of PLSCR1 Tyr69 and Tyr74 was associated with its nuclear translocation and binding to STAT3, we assessed the effect of PLSCR1 and PLSCR1-Y69, 74F on STAT1 expression. We expressed PLSCR1 and PLSCR1-Y69, 74F in shPLSCR1-expressing MDA-MB231, MDA- MB468, and HCC1937 cells (**Figure 6B**). Strikingly, exogenous PLSCR1 but not PLSCR1-Y69, 74F expression restored the decreased STAT1 expression in these cells with stable knockdown of PLSCR1 expression (**Figure 6B**). To investigate whether PLSCR1 bound to the STAT1 promoter, we performed ChIP assays in MDA-MB231, MDA-MB468, and HCC1937 cells with exogenous PLSCR1 or PLSCR1-Y69, 74F expression. A dramatic enrichment of wild-type PLSCR1 but not PLSCR1-Y69, 74F mutation in the STAT1 promoter was observed in these cells (**Figure 6C** and **Figure S5C**). These data suggest that phosphorylation of PLSCR1 Tyr69 and Tyr74 is critical for inducing STAT1 expression.

It is well established that STAT1 and c-Myc are direct target genes of STAT3 34, 36. We performed ChIP assays in MDA-MB231, MDA-MB468, and HCC1937 cells with empty vector or knockdown of PLSCR1 and showed that STAT3 was enriched on the STAT1 or c-Myc promoter in these cells validating the specificity of this assay (**Figure 6D-E** and **Figure S5D-F**). To determine whether PLSCR1 enhances STAT3 binding to the STAT1 promoter, we performed ChIP assays in MDA-MB231, MDA-MB468, and HCC1937 cells with empty vector or knockdown of PLSCR1. We found that, compared with vector control, knockdown of PLSCR1 expression significantly reduced STAT3 binding to the promoter of STAT1 (**Figure 6D-E** and **Figure S5D-E**). These data suggest that PLSCR1 contributes to STAT3 binding to the STAT1 promoter.

A recent study showed that STAT1 promoted breast cancer progression by increasing CSC properties (Qadir *et al*., 2017). Given the association of PLSCR1 with STAT1, we examined the effect of PLSCR1 on tumorsphere formation. As expected, knockdown of PLSCR1 expression significantly suppressed tumorsphere formation in MDA-MB231 and MDA-MB468 cells, whereas PLSCR1 expression dramatically promoted tumorsphere formation in MCF7 and T47D cells (**Figure 6F-G**). Additionally, we assessed the effect of PLSCR1 expression and enzymatic activity on tumorsphere formation in MCF7 and T47D cells with empty vector, wild-type PLSCR1, or PLSCR1-mut expression; the analysis showed that both PLSCR1 and PLSCR1-mut expression had a similar increase in tumorsphere formation (**Figure S6A-B**). Because breast CSCs are characterized by a CD44^high^/CD24^low^ phenotype 37, 38, we evaluated the potential effect of PLSCR1 on cell population with CD44^high^/CD24^low^ properties using flow cytometry analysis. Similar to the finding in tumorsphere formation, knockdown of PLSCR1 expression led to a remarkable decrease of CD44^high^/CD24^low^ population in MDA-MB231 and MDA-MB468 cells, whereas PLSCR1 expression resulted in a dramatic increase of CD44^high^/CD24^low^ population in MCF7 and T47D cells (**Figure 6H-I** and **Figure S6C-D**). These data support the critical role of PLSCR1 in increasing breast cancer stemness.

### PLSCR1 is required for tumorigenicity and metastasis of breast cancer

**Figure 7:**
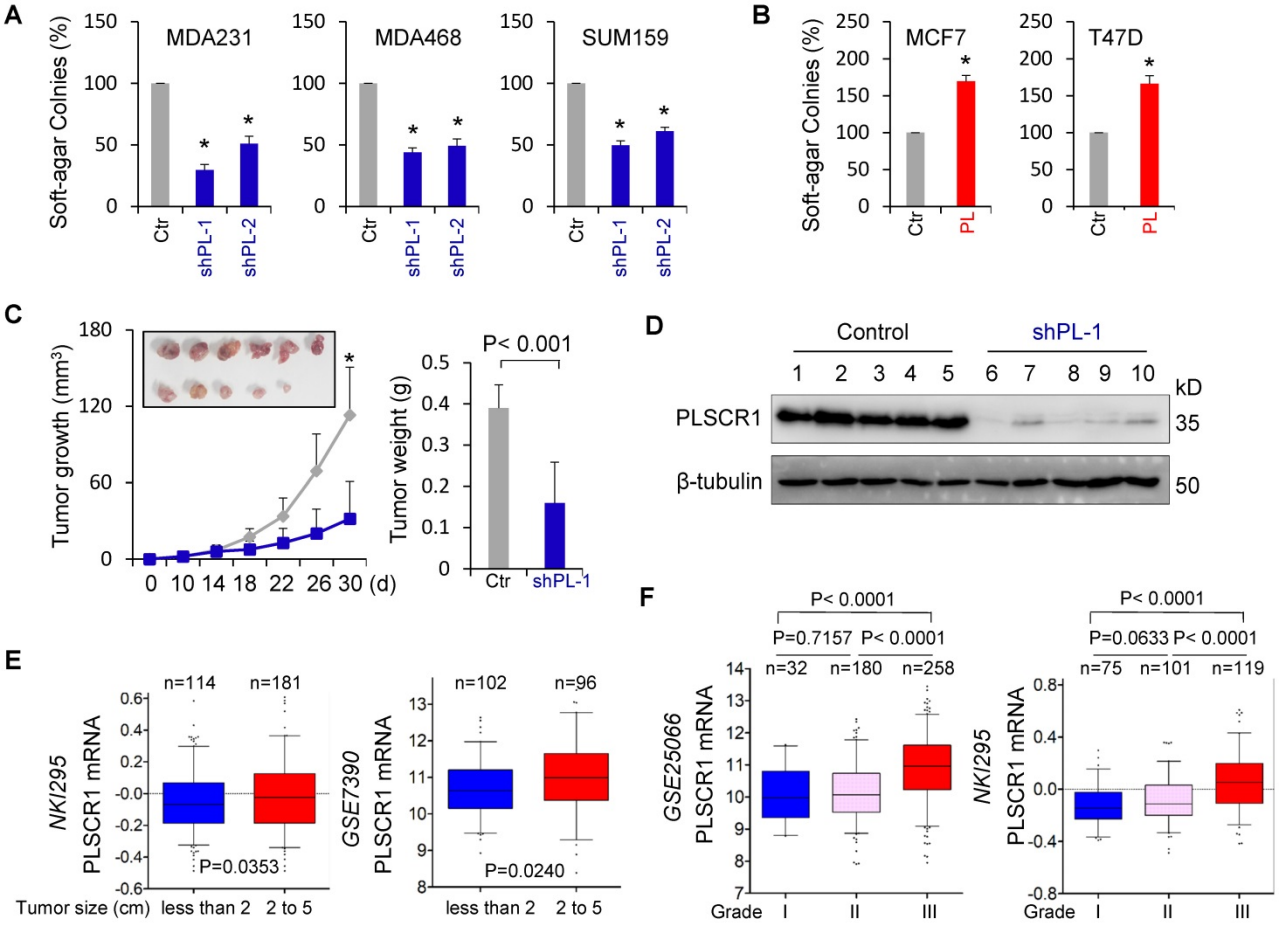
** Knockdown of PLSCR1 expression suppresses tumorigenicity *in vitro* and *in vivo*. (A-B)** Soft-agar assay was performed using MDA-MB231, MDA-MB468, and SUM159 cells with stable empty vector or knockdown of PLSCR1 expression (A) as well as MCF7 and T47D cells with stable empty vector or PLSCR1 expression (B). Data are presented as the percentage of vector cell lines (mean ± SD in three separate experiments). *p< 0.01 by Student's t-test. **(C-D)** MDA-MB231 cells with stable empty vector or knockdown of PLSCR1 expression were injected into the mammary fat pad of SCID mice. Tumor growth (C, left panel) was measured every two days. Tumor weights (C, right panel) were recorded. The expression of PLSCR1 was analyzed by Western blotting in tumor samples removed from two groups of mice (D). Data are presented as mean ± SEM from six mice. *p< 0.05. **(E)** Box-plots indicate PLSCR1 expression in different tumor sizes of breast cancer from NKI295 and GSE7390 datasets. Comparisons are made using the two-tailed Student's t-test. **(F)** Box-plots indicate PLSCR1 expression in different histological grades of breast cancer from GSE25066 and NKI295 datasets. Comparisons between two groups are made using the two-tailed Student's t-test.

CSCs possess highly tumorigenic and metastatic properties 39-41. We first determined the effect of PLSCR1 expression on the *in vitro* tumorigenicity using the soft-agar assay. Knockdown of PLSCR1 expression led to a remarkable decrease of colonies in MDA-MB231, MDA-MB468, and SUM159 cells, whereas PLSCR1 expression resulted in an apparent increase of colony-formation in MCF7 and T47D cells (**Figure 7A-B**). We also evaluated the effect of PLSCR1 expression and enzymatic activity on colony-formation in MCF7 and T47D cells with empty vector, wild-type PLSCR1 or PLSCR1-mut expression and observed a similar increase in colony-formation between PLSCR1 and PLSCR1-mut expression (**Figure S7A**). Next, we tested the* in vivo* tumorigenicity using tumor xenograft models. Markedly, MDA-MB231 cells with knockdown of stable PLSCR1 expression led to reduced tumor growth *in vivo* (**Figure 7C**). Western blotting analysis of tumor samples showed a dramatic decrease in PLSCR1 expression when it was knocked down (**Figure 7D**), which was consistent with the results in cell lines, showing similar inhibitory effects *in vitro* and *in vivo*.

To explore the clinical implications of PLSCR1 expression for breast cancer progression, we first evaluated the association of PLSCR1 expression with tumor size in NKI295 and GSE7390 datasets. Patients were separated into two groups based on the primary tumor size. Significantly, high PLSCR expression was correlated with a larger tumor size (**Figure 7E**). We then assessed the association of PLSCR1 expression with histological grades of breast tumors in GSE25066, NKI295, GSE7390, GSE22358, and MEBTABRIC datasets in which tumors had the malignancy grading scores. Patients were divided into three groups according to breast tumor grades. The analysis showed that PLSCR1 was predominantly expressed in high grade, especially in Grade 3 tumors (**Figure 7F** and **Figure S7B**).

**Figure 8:**
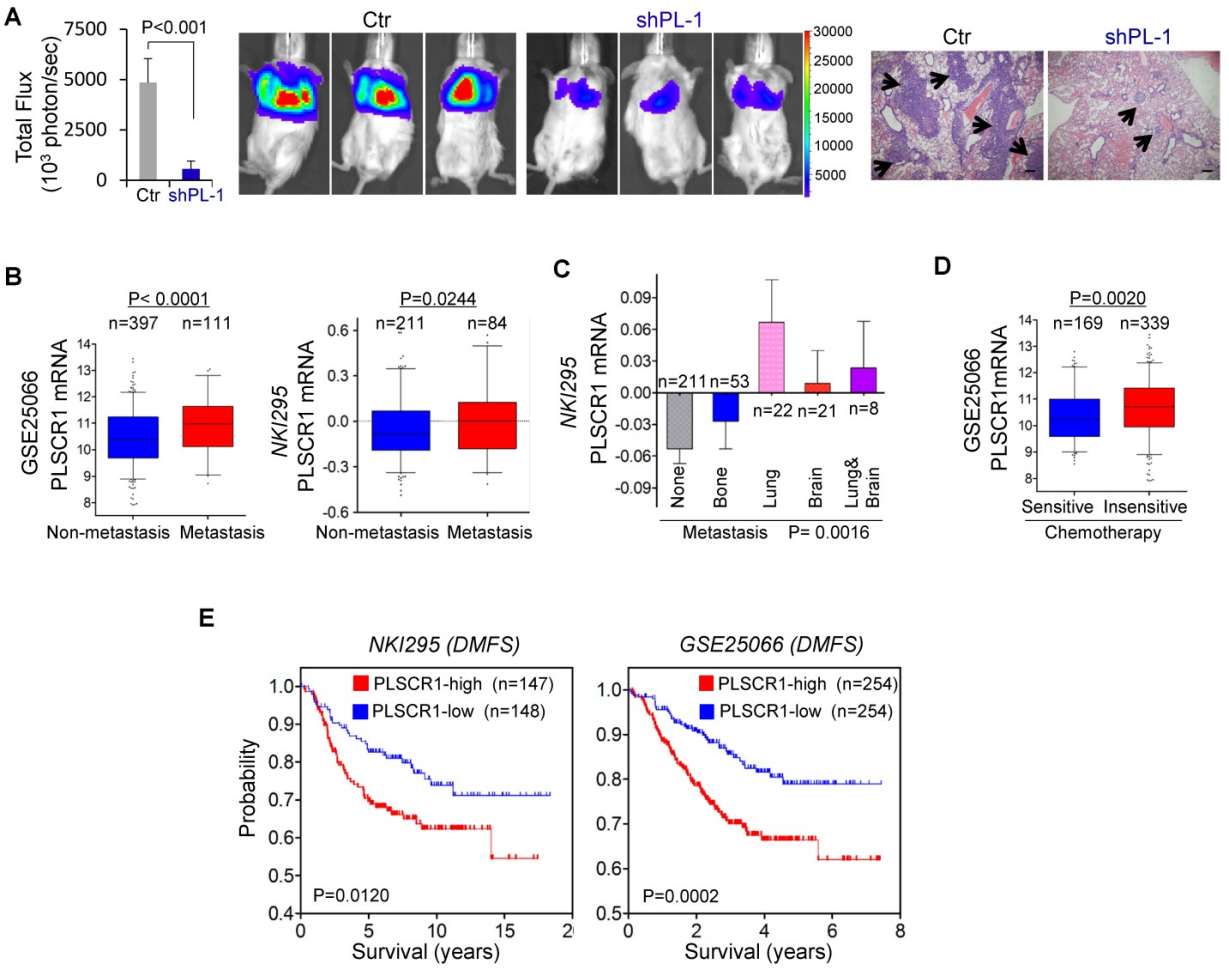
** Knockdown of PLSCR1 expression suppresses metastasis *in vivo,* and elevated PLSCR1 predicts poor clinical outcomes. (A)** MDA-MB231 cells with stable empty vector or knockdown of PLSCR1 expression were injected into SCID mice via the tail vein. After 30 days, lung metastases were quantified by measuring photon flux (mean of 6 animals ± SEM) (left). Three representative mice from each group are presented (middle). Lung metastatic nodules were stained with hematoxylin and eosin. The arrowheads indicate lung metastases. Scale bar = 200 μm (right). **(B)** Analysis of PLSCR1 expression in breast cancer patients with or without metastasis from GSE25066 and NKI295 datasets.** (C)** Analysis of the NKI295 dataset for the association of PLSCR1 expression with the metastatic tendency of primary breast tumors. **(D)** Analysis of the GSE25066 dataset for the association of PLSCR1 expression with chemotherapy sensitivity.** (E)** Kaplan-Meier survival analysis for DMFS of patients in NKI295 and GSE25066 datasets according to PLSCR1 expression status. The p-value is determined using the log-rank test.

Because PLSCR1 was associated with tumor cell migration, invasion, and stemness (**Figure 2C-D** and **Figure 6F-I**), it might also be important for breast cancer metastasis. Due to the inhibitory effect of PLSCR1 knockdown on tumor growth and the metastatic propensity of BLBC, we evaluated the role of PLSCR1 in tumor metastasis using a xenograft metastasis model in which MDA-MB231 cells with stable empty vector or knockdown of PLSCR1 were used to generate pulmonary metastases. Strikingly, knockdown of PLSCR1 expression inhibited lung metastasis in the mouse model (**Figure 8A**). We then examined the clinical relevance of this finding by analyzing the correlation of PLSCR1 expression with lymph node metastasis in the GSE25066 dataset and found that primary tumors with high PLSCR1 expression had more lymph node metastases (**Figure S8A**). We also determined the association of PLSCR1 expression with metastasis in GSE25066 and NKI295 datasets and observed a relatively higher probability of metastasis in tumors with high PLSCR1 expression than those with low PLSCR1 expression (**Figure 8B**). Subsequently, we assessed the relationship between PLSCR1 expression and metastatic sites in the NKI295 dataset. Consistent with the metastatic tendency of BLBC, primary tumors with lung and/or brain metastasis had high PLSCR1 expression (**Figure 8C**). These data suggest that PLSCR1 is important for BLBC cell metastases.

Having identified the critical roles of PLSCR1 in breast cancer, we determined the association of PLSCR1 expression with chemotherapy sensitivity in the GSE25066 dataset in which patients with breast cancer received chemotherapy including sequential taxane and anthracycline-based regimens. Remarkably, tumors with chemotherapy resistance were observed to have high PLSCR1 expression (**Figure 8D**). We then evaluated the correlation of PLSCR1 expression with patient survival in NKI295 and GSE25066 datasets by Kaplan-Meier survival analysis 22, 23. Patients were separated into two groups according to PLSCR1 expression, with high PLSCR1 expression having shorter overall survival (OS), relapse-free survival (RFS), and distant metastasis- free survival (DMFS) (**Figure 8E** and **Figure S8B**). These clinical validations support the critical role of PLSCR1 in breast cancer aggressiveness.

## Discussion

In this study, we report that nuclear translocation of PLSCR1 contributes to the tumorigenic and metastatic ability of BLBC cells and elucidate important underlying mechanisms. Our study provides several new insights into the critical roles of nuclear PLSCR1 in BLBC.

### Nuclear PLSCR1 activates STAT1-mediated stemness of breast cancer cells

Accumulating studies have shown that PLSCR1 may not be a true scramblase due to its unusual features such as low molecular weight, a single transmembrane domain, very low rate of phospholipid scrambling and no alteration of phospholipid scrambling in PLSCR1-mutant mice, suggesting other roles of PLSCR1 within the cells 3, 4, 7. This notion is supported by our data that there was no significant difference in controlling tumor cell proliferation, tumorsphere formation, colony formation, migration, and invasion between wild-type PLSCR1 and catalytically inactive PLSCR1 mutant, indicating that catalytic activity of PLSCR1 might not be required for breast tumorigenesis.

A previous report demonstrated that PLSCR1 binds to the promoter of the IP3R1 gene 8, suggesting that PLSCR1 may either directly activate transcription of targeted genes or enhance the other transcription factors. We showed the complex formation of PLSCR1 with STAT3, which was recruited to the promoter of STAT1, thereby enhancing STAT3-mediated transactivation of STAT1. Double mutation of Tyr 69/74 significantly reduced PLSCR1 binding to STAT3 and the STAT1 promoter, supporting the critical role of phosphorylation of PLSCR1 Tyr 69/74 in STAT3-induced STAT1 transactivation. Collectively, our study has identified a mechanism to support the notion that PLSCR1 regulates the expression of target genes by enhancing relevant transcription factors.

BLBC is characterized by the expression of basal/myoepithelial cell markers and identified as a subtype of breast cancer that might stem from undifferentiated stem cells 42. This subtype is usually triple-negative for ER, PR, and HER2 expression; lack of these receptors often causes a fatal clinical outcome. Indeed, BLBC possesses more CSC properties than the other breast cancer subtypes 37, 43-49. Previous reports have shown that STAT1, as a tumor activator, promotes breast cancer progression by conferring CSC properties on cancer cells 11, 12. Consistent with this concept, PLSCR1 expression resulted in increased CSC properties by promoting transactivation of STAT1 in BLBC, implying the critical role of PLSCR1 in controlling the viability of CSCs, which are implicated in mediating tumor initiation and metastasis 39-41. Indeed, our results showed that knockdown of PLSCR1 expression dramatically suppressed tumorigenicity and metastasis *in vitro* and *in vivo*, supporting the instrumental role of PLSCR1 in STAT1-mediated aggressiveness of BLBC cells.

### Nuclear translocation of PLSCR1 is mediated by its phosphorylation

Palmitoylation of PLSCR1 regulates its trafficking to the cell membrane or the nucleus. When PLSCR1 is not palmitoylated, it can be transported into the nucleus 6. Our data showed that following EGF treatment, non-palmitoylated mutant promoted nuclear translocation of PLSCR1, whereas Y69, 74F mutant efficiently blocked non-palmitoylated mutation-induced nuclear translocation of PLSCR1. These findings suggest that non-palmitoylation of PLSCR1 is necessary but not sufficient for mediating nuclear translocation, and phosphorylation of PLSCR1 Tyr 69/74 is required for the nuclear translocation of the non-palmitoylated protein.

### PLSCR1 represents a potential prognostic indicator and therapeutic target for BLBC

Because of the association of PLSCR1 with breast cancer aggressiveness, it was important to assess the possibility of PLSCR1 as a prognostic indicator for breast cancer patients. We have identified several factors that predict patient prognosis, including (1) breast cancer subtypes: PLSCR1 expression is elevated in BLBC; (2) tumor size: high PLSCR1 expression is associated with larger tumor size; (3) tumor grade: high PLSCR1 expression is correlated with higher tumor grade; (4) tumor metastasis: high PLSCR1 expression has a significantly higher probability of regional lymph node and distant metastasis, and metastatic dissemination to the brain and lungs that is consistent with the metastatic propensity of BLBC; (5) chemotherapy: high PLSCR1 expression is correlated with chemotherapy resistance in breast cancer patients; (6) survival rate: high PLSCR1 expression predicts poor survival in breast cancer patients. These findings strongly support PLSCR1 as the potential biomarker for breast cancer patients.

Our study demonstrated a tight association of nuclear PLSCR1 with increased CSC properties caused by the transactivation of STAT1 in BLBC. This observation might be especially significant because traditional cancer therapies are often ineffective against the minor population of CSCs that regenerate tumors (Gupta *et al*., 2009). Our study has also established that phosphorylation of PLSCR1 Tyr 69/74 plays an instrumental role in the proliferation and stemness of breast cancer cells by promoting its nuclear translocation, interaction with STAT3 and subsequent binding to the STAT1 promoter. It has been reported that PLSCR1 Tyr 69/74 is a substrate of c-Abl tyrosine kinase 50, implying that both phosphorylation sites and the tyrosine kinase might be potential targets in the clinical treatment of BLBC.

## Conclusions

To summarize, our study has provided several mechanistic and therapeutic insights into the crucial roles of PLSCR1 in BLBC progression. Most importantly, our results afford a link between the nuclear PLSCR1-mediated transactivation of STAT1 and BLBC aggressiveness. Thus, our study is expected to improve the prospects of developing prevention strategies and effective treatment for the aggressive subtype of breast cancer.

## Abbreviations

PL (PLSCR1): phospholipid scramblase 1
PL-mut: PLSCR1 loss of scramblase activity
BLBC: basal-like breast cancer
STAT1: signal transducer and activator of transcription 1
STAT3: signal transducer and activator of transcription 3
p-STAT1: phosphorylated STAT1
p-STAT3: phosphorylated STAT3
EGF: epidermal growth factor
hIL-6: recombinant human Interleukin-6
CSC: cancer stem cell
CCK8: cell counting Kit-8
co-IP: co-immunoprecipitation
ChIP: chromatin immunoprecipitation
RT-PCR: reverse transcription-polymerase chain reaction
DMFS: distant metastasis-free survival
OS: overall survival
RFS: relapse-free survival
