# Supplementary material for: Nuclear translocation of PLSCR1 activates STAT1 signaling in basal-like breast cancer
Source: Theranostics. 2020 Mar 25;10(10):4644–58. doi: 10.7150/thno.43150 (PMC7150476; doi:10.7150/thno.43150)
Supplement: Supplementary file 1 — Supplementary figures and tables. [file thnov10p4644s1.pdf]

Supplementary Materials

Supplementary Figure Legends

Figure S1

(A) Box-plots indicate PLSCR1 mRNA expression in breast cancer from the GSE7390 and NKI295 datasets.

(B) Box-plots indicate PLSCR1 mRNA expression in luminal and BLBC cell lines from the GSE16732 dataset.

Figure S2

(A) Expression of PLSCR1 was examined by Western blotting in SUM159 cells with stable empty vector or knockdown of PLSCR1 expression as well as shPLSCR1-expressing SUM159 cells with stable empty vector, PLSCR1 or PLSCR1-mut (no enzymatic activity) expression.

(B) Cell growth for SUM159 cells with stable empty vector or knockdown of PLSCR1 expression as well as shPLSCR1-expressing SUM159 cells with stable empty vector, PLSCR1 or PLSCR1-mut expression (left panel), and T47D cells with stable empty vector, PLSCR1 or PLSCR1-mut expression (right panel) was analyzed by cell counting kit-8 assay for a period of 120 hours. Data are presented as a percentage over control cells (mean ± SD in three separate experiments). *p< 0.05 by Student's t-test.

(C) Migratory ability (left) and invasiveness (right) of SUM159 cells with stable empty vector or knockdown of PLSCR1 expression as well as shPLSCR1-expressing SUM159 cells with stable empty vector, PLSCR1 or PLSCR1-mut expression were analyzed. The percentage of migratory and invasive cells is presented in the bar graph (mean ± SD in three separate experiments). *p< 0.01 by Student's t-test.

Figure S3

(A) Expression of PLSCR1 was measured by immunofluorescent staining in HCC1937 cells treated with or without EGF (100 ng/ml). Nuclei are visualized with DAPI (blue). Scale bar = 20 μm (right).

(B) Expression of PLSCR1 was examined by Western blotting in HCC1937 cells treated with or without EGF (100 ng/ml) for a period of 0, 1 or 3 hours.

(C) Expression and localization of PLSCR1 were measured by immunofluorescent staining in shPLSCR1-

expressing HCC1937 cells with stable PLSCR1, PLSCR1-Y69, 74F, PLSCR1 (184-189) CA or PLSCR1 (184-189) CA-Y69, 74F expression following treatment with EGF (100 ng/ml) (left panel). Nuclei were visualized with DAPI (blue). Scale bar = 20 μm (right). Nuclear-cytoplasmic staining percentage is shown in right panel (mean ± SD in three separate experiments). *p< 0.05 by Student's t-test.

Figure S4

(A) A stable shPLSCR1-expressing MDA-MB231 cells with PLSCR1-2Flag expression was established, and PLSCR1 complex was isolated by Flag affinity columns. The bound proteins such as EGFR and FYN were identified by Mass Spectrometry.

(B) PLSCR1-2Flag and EGFR-6His were co-expressed in HEK293T and MDA-MB231 cells. Following immunoprecipitation, the bound EGFR and PLSCR1 were examined by Western blotting.

Figure S5

(A and B) Expression of STAT3 and STAT1 was examined by Western blotting in MDA-MB231 and SUM159 cells with stable empty vector or knockdown of STAT3 expression (A) as well as MCF7 and T47D cells with stable empty vector or STAT3 expression (B).

(C) The association of wild-type PLSCR1 or PLSCR1-Y69, 74F with the STAT1 promoter in HCC1937 cells was analyzed by ChIP following treatment with or without EGF (100 ng/ml).

(D and E) ChIP analysis for binding of STAT3 to the STAT1 promoter in HCC1937 cells with stable empty vector or knockdown of PLSCR1 expression following treatment with hIL-6 (100 ng/ml) by either semi-quantitative RT-PCR (D) or quantitative real-time PCR (E). Results from three independent experiments are presented (mean ± SD from three separate experiments). *p< 0.01 by Student's t-test.

(F) ChIP analysis for binding of STAT3 to the c-Myc promoter in MDA-MB231, MDA-MB468 and HCC1937 cells with stable empty vector or knockdown of PLSCR1 expression by semi-quantitative RT-PCR.

Figure S6

(A) Expression of PLSCR1 was examined by Western blotting in MCF7 and T47D cells with stable empty vector, PLSCR1 or PLSCR1-mut (no enzymatic activity) expression.

(B) Tumorsphere-formation of MCF7 and T47D cells with stable empty vector, PLSCR1 or PLSCR1-mut expression was measured. Data are shown as a percentage of control cell lines (mean ± SD in three separate experiments). *p< 0.01 by Student's t-test.

(C and D) Population of CSCs ($CD44^{high}/CD24^{low}$) were analyzed by flow cytometry in MDA-MB231 and MDA-MB468 cells with stable empty vector or knockdown of PLSCR1 expression (C) as well as MCF7 and T47D cells with stable empty vector or PLSCR1 expression (D). Representative images were shown.

Figure S7

(A) Soft-agar assay was performed using MCF7 and T47D cells with stable empty vector, PLSCR1 or PLSCR1-mut expression. Data are presented as a percentage of vector cell lines (mean ± SD in three separate experiments). *p< 0.01 by Student's t-test.

(B) Box-plots indicate PLSCR1 expression in different histological grades of breast cancer from GSE7390, GSE22358 and MEBTABRIC datasets. Comparisons between two groups are made using the two-tailed Student's t-test.

Figure S8

(A) Box-plots indicate PLSCR1 expression in different status of lymph node metastases of breast cancer from the GSE25066 dataset. Comparisons between two groups are made using the two-tailed Student’s t-test.

(B) Kaplan-Meier survival analysis for OS and RFS of patients in the NKI295 dataset according to PLSCR1 expression status. The p value is determined using the log-rank test.

Supplementary Figures

Figure S1

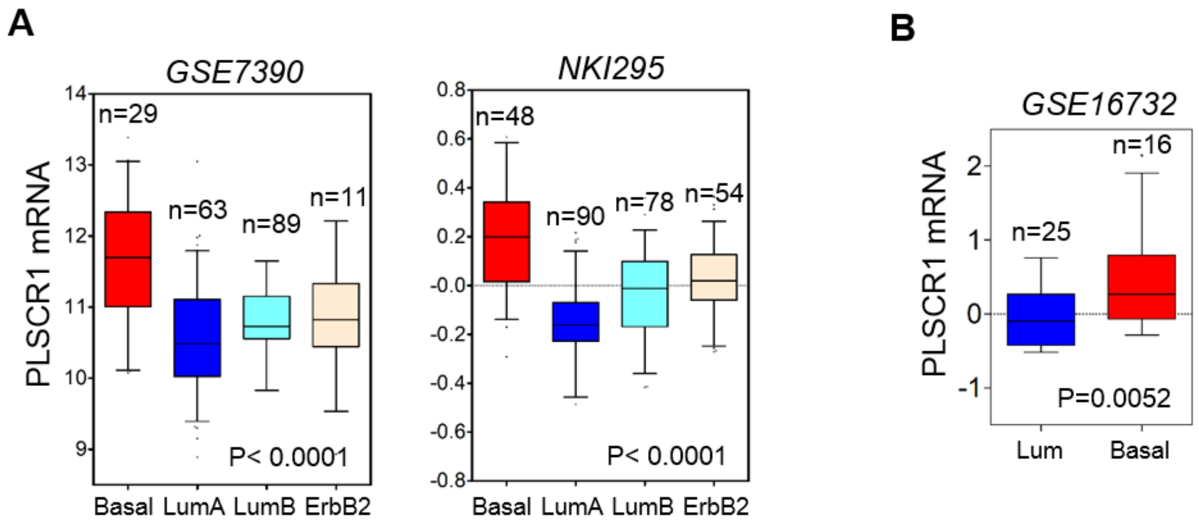

Figure S2

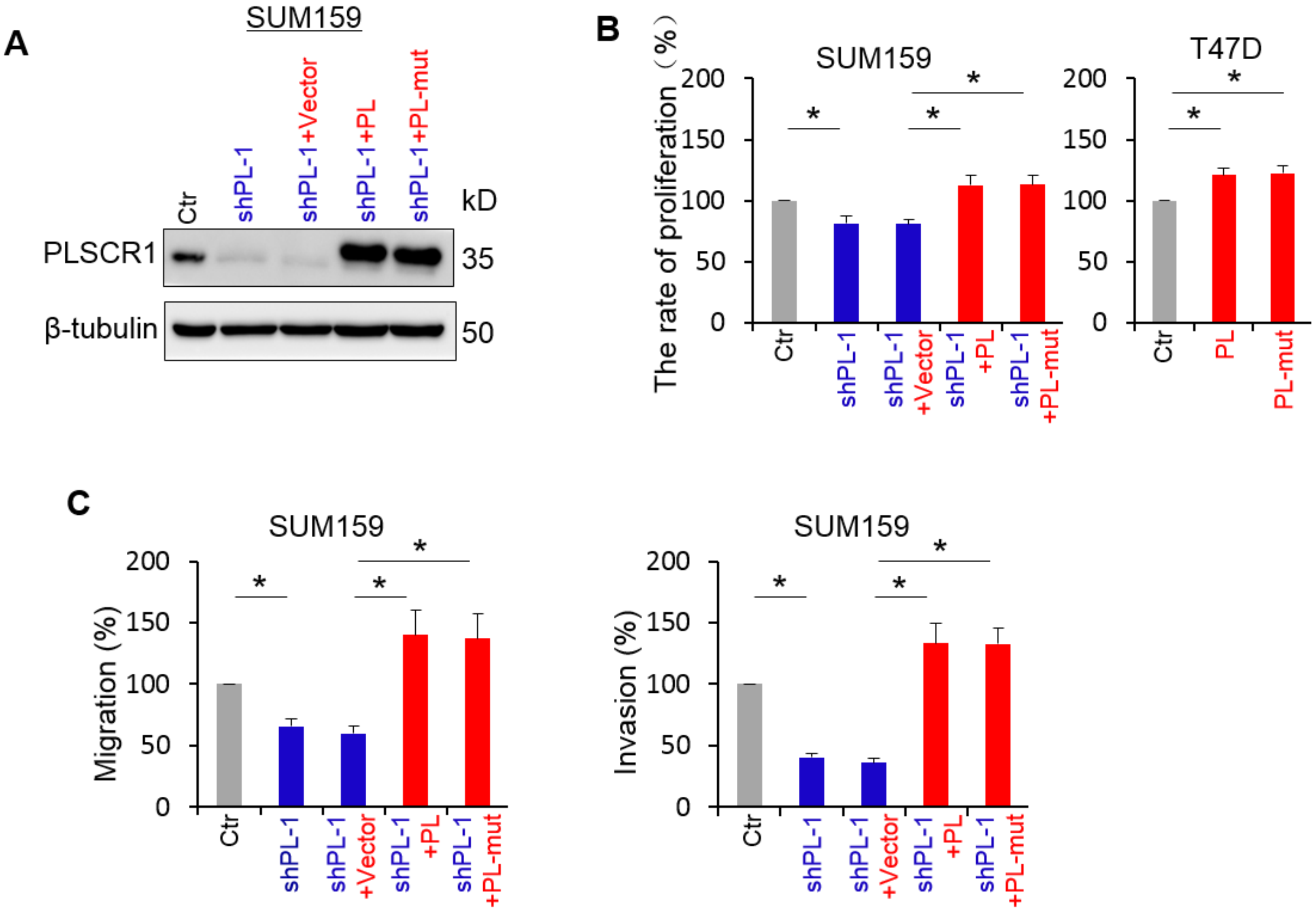
A
SUM159
Ctr
shPL-1
shPL-1+Vector
shPL-1+PL
shPL-1+PL-mut
kD
PLSCR1
35
β-tubulin
50
B
SUM159
The rate of proliferation（%）
200
150
100
50
0
Ctr
shPL-1
shPL-1 +Vector
shPL-1 +PL
shPL-1 +PL-mut
T47D
Ctr
PL
PL-mut
C
SUM159
Migration (%)
Ctr
shPL-1
shPL-1 +Vector
shPL-1 +PL
shPL-1 +PL-mut
SUM159
Invasion (%)
Ctr
shPL-1
shPL-1 +Vector
shPL-1 +PL
shPL-1 +PL-mut

Figure S3

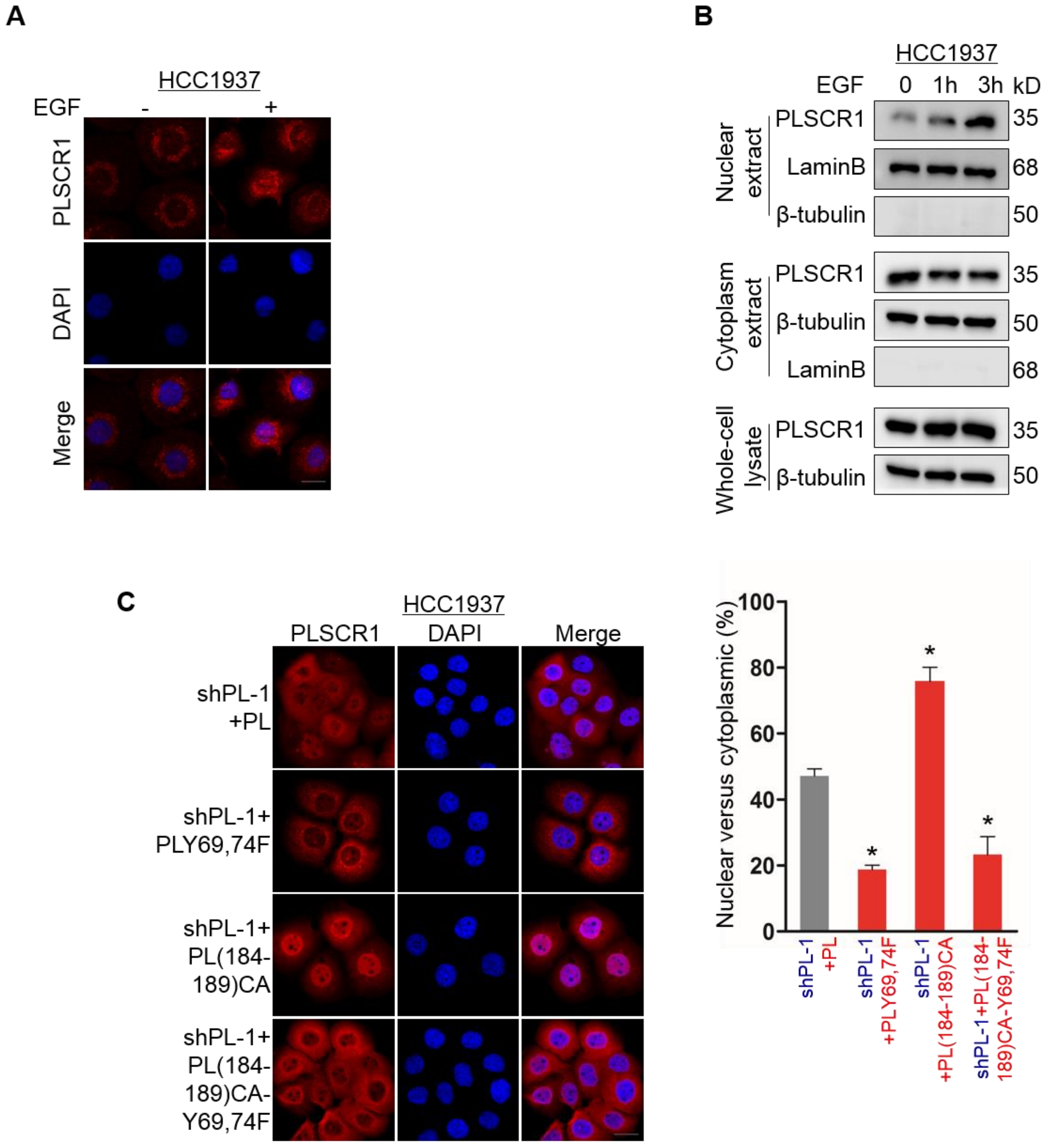
A
HCC1937
EGF
-
+
PLSCR1
DAPI
Merge
B
HCC1937
EGF
0
1h
3h
kD
Nuclear extract
PLSCR1
35
LaminB
68
β-tubulin
50
Cytoplasm extract
PLSCR1
35
β-tubulin
50
LaminB
68
Whole-cell lysate
PLSCR1
35
β-tubulin
50
C
HCC1937
PLSCR1
DAPI
Merge
shPL-1 +PL
shPL-1+ PLY69,74F
shPL-1+ PL(184-189)CA
shPL-1+ PL(184-189)CA-Y69,74F
Nuclear versus cytoplasmic (%)
100
80
60
40
20
0
*
shPL-1 +PL
shPL-1 +PLY69,74F
shPL-1 +PL(184-189)CA
shPL-1+PL(184-189)CA-Y69,74F

Figure S4

A

IP mass spectrometry analysis report

163	213_1	sp|Q9HAV4|XPO5_HUMAN	3.093395
164	8_1	sp|Q9Y6N5|SQOR_HUMAN	4.355527
165	396_1	sp|P62826|RAN_HUMAN	17.42211
166	289_1	sp|P00533|EGFR_HUMAN	4.355527
167	104_1	sp|Q99720|SGMR1_HUMAN	4.355527

10	64_2	sp|P08238|HS90B_HUMAN	21.17140348
11	240_1	sp|P53618|COPB_HUMAN	11.67810349
12	205_1	sp|Q9Y262|EIF3L_HUMAN	31.1522134
13	22_1	sp|P06241|FYN_HUMAN	2.871024801
14	20_1	sp|P04181|OAT_HUMAN	8.711053888

B

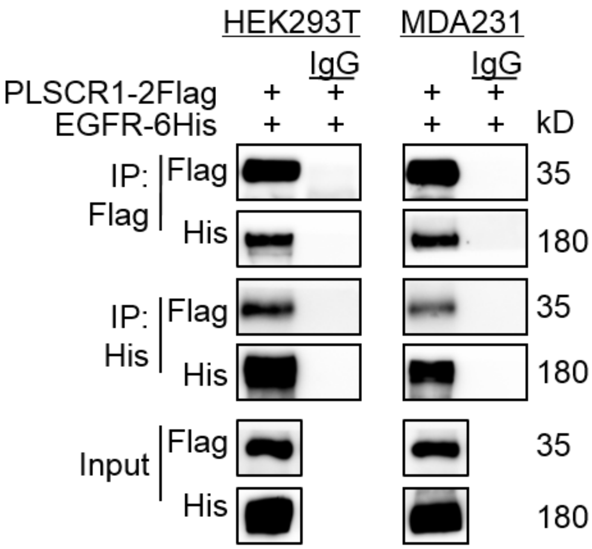

Figure S5

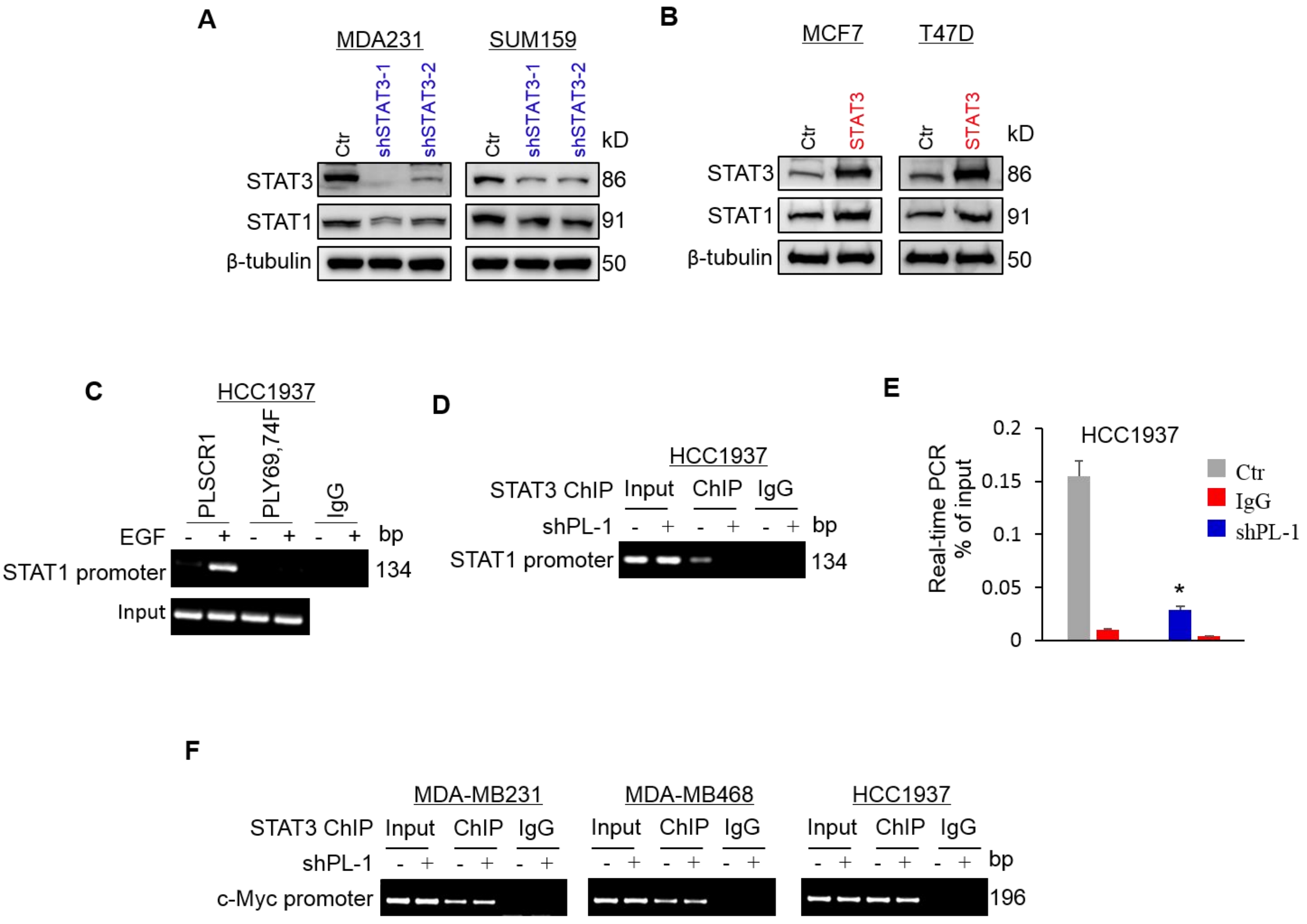
A
MDA231
SUM159
Ctr
shSTAT3-1
shSTAT3-2
kD
STAT3
STAT1
β-tubulin
86
91
50
B
MCF7
T47D
Ctr
STAT3
kD
STAT3
STAT1
β-tubulin
86
91
50
C
HCC1937
PLSCR1
PLY69,74F
IgG
EGF
- + - + - +
bp
STAT1 promoter
134
Input
D
HCC1937
STAT3 ChIP
Input
ChIP
IgG
shPL-1
- + - + - +
bp
STAT1 promoter
134
E
HCC1937
Real-time PCR
% of input
0.2
0.15
0.1
0.05
0
Ctr
IgG
shPL-1
*
F
MDA-MB231
MDA-MB468
HCC1937
STAT3 ChIP
Input
ChIP
IgG
shPL-1
- + - + - +
bp
c-Myc promoter
196

Figure S6

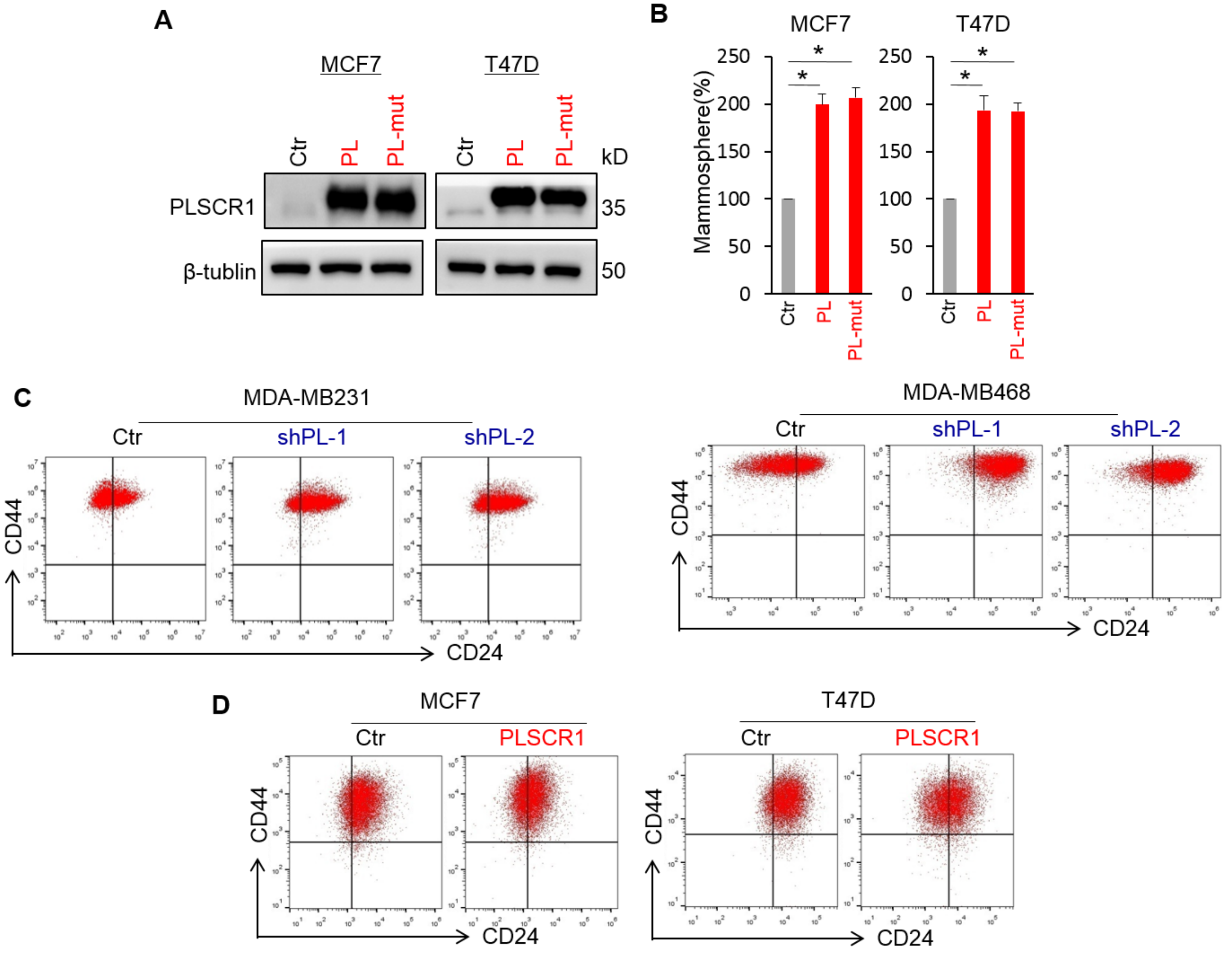
A
MCF7
T47D
Ctr
PL
PL-mut
kD
PLSCR1
35
β-tublin
50
B
Mammosphere(%)
250
200
150
100
50
0
*
C
MDA-MB231
shPL-1
shPL-2
CD44
CD24
MDA-MB468
D
PLSCR1

Figure S7

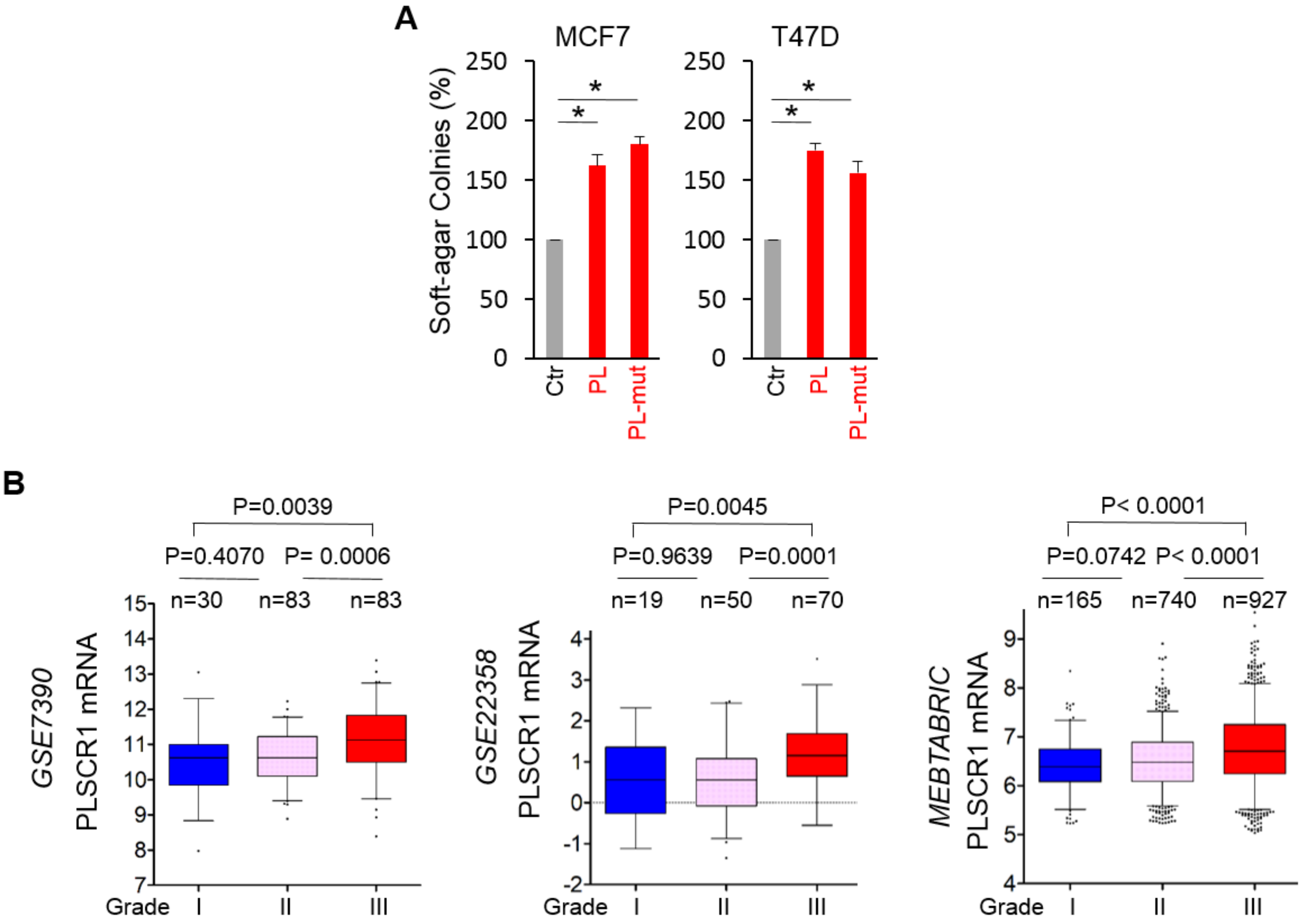
A
MCF7
T47D
Soft-agar Colnies (%)
250
200
150
100
50
0
*
*
Ctr
PL
PL-mut
B
P=0.0039
P=0.4070
P= 0.0006
n=30
n=83
n=83
GSE7390
PLSCR1 mRNA
Grade
I
II
III
P=0.0045
P=0.9639
P=0.0001
n=19
n=50
n=70
GSE22358
PLSCR1 mRNA
P< 0.0001
P=0.0742
P< 0.0001
n=165
n=740
n=927
MEBTABRIC
PLSCR1 mRNA

Figure S8

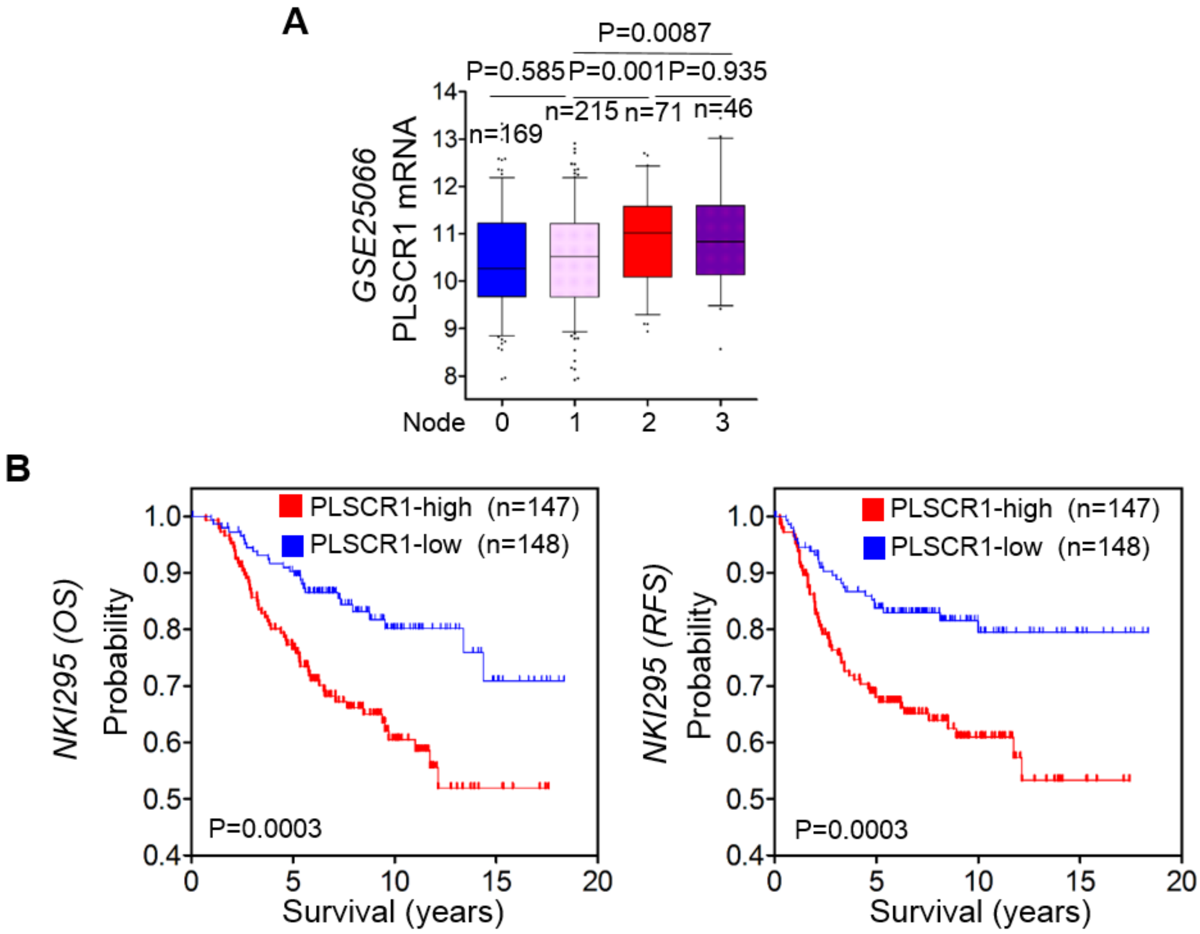
A
P=0.0087
P=0.585
P=0.001
P=0.935
n=169
n=215
n=71
n=46
GSE25066
PLSCR1 mRNA
Node
0
1
2
3
B
PLSCR1-high (n=147)
PLSCR1-low (n=148)
NKI295 (OS)
Probability
P=0.0003
Survival (years)
PLSCR1-high (n=147)
PLSCR1-low (n=148)
NKI295 (RFS)
Probability
P=0.0003
Survival (years)
